# Earth-abundant Zn–dipyrrin chromophores for efficient CO_2_ photoreduction

**DOI:** 10.1093/nsr/nwae130

**Published:** 2024-04-01

**Authors:** Song Guo, Fu-Gui Zeng, Xiao-Di Li, Kai-Kai Chen, Ping Wang, Tong-Bu Lu, Zhi-Ming Zhang

**Affiliations:** Institute for New Energy Materials and Low Carbon Technologies, School of Materials Science & Engineering, Tianjin University of Technology, Tianjin 300384, China; Institute for New Energy Materials and Low Carbon Technologies, School of Materials Science & Engineering, Tianjin University of Technology, Tianjin 300384, China; Institute for New Energy Materials and Low Carbon Technologies, School of Materials Science & Engineering, Tianjin University of Technology, Tianjin 300384, China; Institute for New Energy Materials and Low Carbon Technologies, School of Materials Science & Engineering, Tianjin University of Technology, Tianjin 300384, China; Institute for New Energy Materials and Low Carbon Technologies, School of Materials Science & Engineering, Tianjin University of Technology, Tianjin 300384, China; Institute for New Energy Materials and Low Carbon Technologies, School of Materials Science & Engineering, Tianjin University of Technology, Tianjin 300384, China; Institute for New Energy Materials and Low Carbon Technologies, School of Materials Science & Engineering, Tianjin University of Technology, Tianjin 300384, China

**Keywords:** Zn complex, photocatalysis, CO_2_ reduction, charge transfer, photosensitizer

## Abstract

The development of strong sensitizing and Earth-abundant antenna molecules is highly desirable for CO_2_ reduction through artificial photosynthesis. Herein, a library of Zn–dipyrrin complexes (**Z-1**−**Z-6**) are rationally designed via precisely controlling their molecular configuration to optimize strong sensitizing Earth-abundant photosensitizers. Upon visible-light excitation, their special geometry enables intramolecular charge transfer to induce a charge-transfer state, which was first demonstrated to accept electrons from electron donors. The resulting long-lived reduced photosensitizer was confirmed to trigger consecutive intermolecular electron transfers for boosting CO_2_-to-CO conversion. Remarkably, the Earth-abundant catalytic system with **Z-6** and Fe-catalyst exhibits outstanding performance with a turnover number of >20 000 and 29.7% quantum yield, representing excellent catalytic performance among the molecular catalytic systems and highly superior to that of noble-metal photosensitizer Ir(ppy)_2_(bpy)^+^ under similar conditions. Experimental and theoretical investigations comprehensively unveil the structure–activity relationship, opening up a new horizon for the development of Earth-abundant strong sensitizing chromophores for boosting artificial photosynthesis.

## Teaser

A library of strong sensitizing Zn-dipyrrin complexes were developed for efficient CO_2_ photoreduction via symmetry breaking charge transfer.

## INTRODUCTION

Excessive greenhouse gas emissions have caused global warming, and CO_2_ concentrations in particular have exceeded 400 ppm on Earth [1,2]. In these circumstances, an appealing strategy has been proposed to construct efficient artificial photosynthetic systems for solar-driven conversion of CO_2_ into fuels, which can reintroduce excess CO_2_ into the carbon cycle in order to realize carbon neutrality [3–5]. In this field, molecular catalytic systems composed of antenna molecules and catalysts have exhibited distinct superiorities in CO_2_ photoreduction due to their well-defined and tunable structures and controllable ground/excited state properties [6–9]. In these systems, the photosensitizer (PS), as a central component for light absorption and electron transfer, has a significant influence on CO_2_ reduction efficiency, but the development of a PS for CO_2_ photoreduction is still in its infancy [7,10]. In this field, Ru(II)/Ir(III) polypyridine complexes have been widely employed as antenna molecules for coupling with various catalysts to drive CO_2_ photoreduction due to their suitable redox potential and supporting photochemical/photophysical properties [4,6,11–16]. For example, Ishitani *et al*. employed Ru(II)/Ir (III) redox PSs and a Re(I) catalyst to efficiently drive CO_2_ photoreduction in the presence of 1,3-dimethyl-2-phenyl-2,3- dihydro-1H-benzo[d]imidazole (BIH) [17,18]. Lau *et al*. achieved high-performance CO_2_-to-CO conversion with turnover numbers (TONs) of >2000 through sensitizing [M(qpy)(OH_2_)_2_]^2+^ (M = Fe, Co) catalysts with the typical Ru(bpy)_3_^2+^ (Ru-1) PS [11,19,20]. In this field, we have proposed a strategy to significantly enhance CO_2_ reduction efficiency by improving the sensitizing ability of the PS, realized by engineering the excited state of Ru(Phen)_3_^2+^ with a pyrenyl functional group [21]. Recently, several attempts have been made to connect Ru(II)/Ir (III) PSs with catalysts by covalent or supramolecular interactions for accelerating electron transfer [22–26]. However, the high cost and ultra-low content of Ru and Ir in Earth's crust have severely impeded the large-scale and long-term applications of these noble-metal PSs [27]. As a result, it is highly desirable but remains a great challenge to develop noble-metal-free PSs for constructing efficient and sustainable photocatalytic systems.

Earth-abundant transition metals, such as Fe, Zn, Cu and Zr, have been widely employed to coordinate with organic ligands to construct functional complexes [28–34]. However, very few of them were explored as PSs to drive CO_2_ photoreduction due to their limitations of poor light-harvesting ability or low photochemical stability. Recently, there has been increasing interest in the search for first-row transition-metal complexes to replace Ru(II)/Ir (III) PSs for sustainable CO_2_ photoreduction. In this field, Cu(I)–phosphine complexes with strong reducing ability were applied to drive CO_2_ photoreduction by coupling Fe, Cu and Mn-based catalysts [35–42]. For example, dimeric Cu complexes with heteroleptic diimine and phosphine ligands were used as the PS to achieve >1000 TON of CO [43]. Very recently, a noble-metal-free catalytic system containing a strong visible-light-absorbing copper–purpurin PS, iron–porphyrin catalyst and BIH was reported to achieve a high TON of 16 100 with a CO yield of 16 μmol [44]. The copper–purpurin PS exhibits much higher performance in CO_2_ photoreduction than other noble-metal-free homogeneous systems. However, such Cu PSs with a special structure are hard to tailor at the molecular level in order to further improve their sensitizing ability. Currently, very limited PSs with more abundant metals have been explored with strong sensitizing ability for boosting CO_2_ reduction [45].

Zn is an abundant element in Earth's crust and the Zn^2+^ ion can be stabilized by various organic ligands to construct photoactive complexes [27,46,47]. It is therefore of great potential interest to construct strong sensitizing Zn PSs for sustainable and efficient CO_2_ reduction. In nature, symmetry-breaking charge transfer (SBCT) usually occurs in the reaction center of purple bacteria, where bacteriochlorophyll dimer is encircled by two approximately identical branches of protein-bound cofactors [48]. Under light irradiation, this SBCT process can produce the location of the electron/hole pair in different chromophores, resulting in a greatly retarded back recombination rate, which can provide a potential pathway for intermolecular electron transfer. Inspired by nature, SBCT may offer a promising strategy toward developing strong sensitizing PSs with more abundant metals. With reference to a biological model, Zn–dipyrrin complexes with symmetric chromophores conform to the structural characteristics for an SBCT effect, and have received considerable attention due to their advantages of strong visible absorption and easy/scalable preparation [49–52]. However, traditional Zn–dipyrrin complexes commonly suffer from loss of excitation energy via phenyl ring rotation at the meso-position of the dipyrrin and excitonic coupling between the nonorthogonal ligands due to their flexible structures. Pioneering works have confirmed that these energy dissipation pathways in Zn PSs could be blocked by enhancing the torsional resistance and keeping an orthogonal configuration [52–54]. As a result, the development of rigid structural Zn complexes with efficient SBCT process represents a promising direction for constructing strong sensitizing Earth-abundant PSs for solar energy utilization. However, such strong sensitizing Zn–dipyrrin complexes have rarely been used to construct artificial photosynthesis systems for highly efficient CO_2_ reduction.

In this contribution, a series of Zn–dipyrrin complexes with two virtually identical and symmetric chromophores were designed and fabricated by introducing anthryl derivatives to engineer the structure of dipyrrole ligands for fundamentally understanding their structure–activity relationship. The Zn–dipyrrin complexes were used for the first time for CO_2_ photoreduction and the influence of the SBCT effect on CO_2_ reduction was first revealed in this work. The charge transfer (CT) states induced via an intramolecular CT process in these Zn PSs were first demonstrated to accept electrons from the BIH to form a long-lived reduced PS. The reduced PS can trigger intermolecular electron transfer to efficiently sensitize [Fe(qpy)(OH_2_)_2_]^2+^ (qpy = 2,2′:6′,2″:6″,2′″-quaterpyridine) (C-1) for boosting CO_2_-to-CO conversion upon visible-light excitation. Impressively, the **Z-6**-based catalytic system exhibited outstanding catalytic performance of >110 μmol CO yield with nearly 100% selectivity, and the TON and quantum yield reached 20 000 and 29.7%, respectively, representing excellent catalytic performance among molecular catalytic systems that was far superior to that of typical noble-metal PSs Ir(bpy)(ppy)^2+^ (**Ir-1**) and Ru(bpy)_3_^2+^ (**Ru-1**) under similar conditions. This work provides a molecular platform to develop a noble-metal-free catalytic system via exploring highly efficient Zn PSs for boosting artificial photosynthesis.

## RESULTS

### Preparation and characterization of Zn–dipyrrin PSs

**Figure 1.**
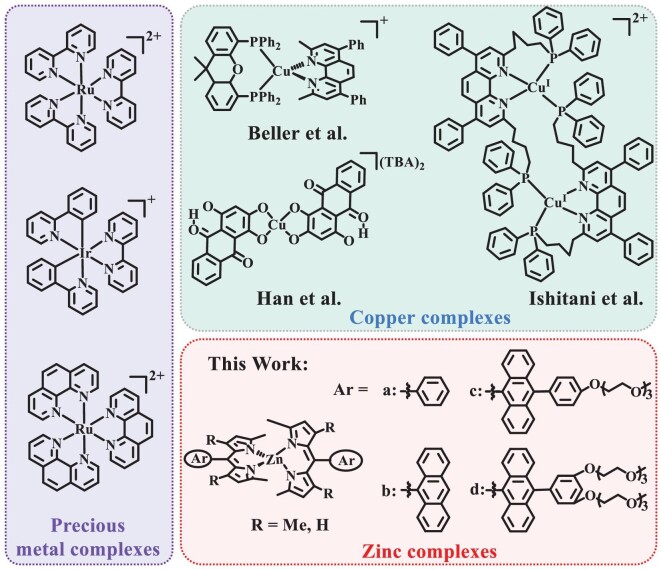
The progress of transition-metal complexes as PSs for CO_2_ reduction.

**Figure 2.**
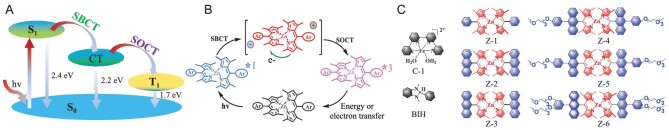
Molecular structures and work mechanism. (A) A simplified Jablonski diagram and (B) the evolution of excited molecular structures illustrating the CT and intersystem crossing (ISC) process. (C) The structures of Zn–dipyrrin PSs, BIH and C-1. S_1_ represents the singlet state; T_1_ represents the triplet state. SOCT represents the spin–orbit charge transfer.

In this work, the dipyrrin ligand in **Z-1** was carefully engineered by introducing anthryl derivatives to increase the torsional resistance in these Zn–dipyrrin complexes of **Z-2, Z-4** and **Z-6** to keep an orthogonal molecular configuration and improve their excited state properties (Figs 1 and 2). **Z-3** and **Z-5**, with flexible structures, were also designed by the removal of methyl as the control PSs. Moreover, **Z-4**−**Z-6** were decorated with polyethylene glycol (PEG) to improve their solubility in aqueous solution. As shown in Supporting Information, Fig. S1, dipyrrin ligands (2, 4, 6, 8 and 19) can be prepared by the reaction of 2,4-dimethyl pyrrole or 2-methyl pyrrole with aromatic aldehyde (1, 3, 5 or 18) [55]. The Zn–dipyrrin complexes **Z-1**−**Z-6** can be easily prepared by stirring the mixture of dipyrrin ligands and zinc acetate at room temperature. The structures of all the target compounds were well characterized by using ^1^H NMR (nuclear magnetic resonance), ^13^C NMR and high-resolution mass spectrometry (Figs S2–S29). The well-defined structures of these Zn PSs can provide ideal molecular platforms to clarify the structure–activity relationship for CO_2_ photoreduction.

### Visible-light-driven CO_2_ reduction

**Figure 3.**
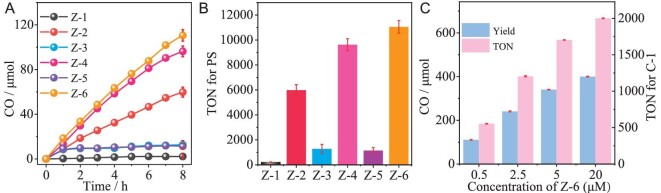
CO_2_ photoreduction. (A) CO generation as a function of irradiation time with different Zn PSs. (B) TON toward PSs. (C) Photocatalytic performance with increasing the concentration of **Z-6**. Catalytic conditions: 0.5 μM PS, 10 μM C-1, 20 mM BIH in 20 mL mixed solvent of CH_3_CN/H_2_O. Under CO_2_ atmosphere, Xenon lamp with 420-nm filter as light source.

To the best of our knowledge, Zn–dipyrrin complexes have never been used for photocatalytic CO_2_ reduction and the influence of the CT effect on CO_2_ reduction was first explored here. In this work, these Zn PSs were used to couple with the Fe-based catalyst (C-1) to construct noble-metal-free catalytic systems for CO_2_ reduction. Photocatalytic experiments were carried out in a mixed solvent of H_2_O/CH_3_CN in the presence of BIH upon irradiation using 300 W Xenon (>420 nm) (Fig. 3, Fig. S30 and Tables S1–S3). As shown in Fig. 3, the sensitizing ability of these catalytic systems with different Zn PSs were in the order of **Z-6** > **Z-4** > **Z-2** > **Z-3** ≈ **Z-5** > **Z-1**. Remarkably, the CO yield and TON for **Z-6** can reach as high as 110.6 μmol and 11 056, respectively, which is >50 times higher than that for **Z-1** with a CO yield of 2.2 μmol (TON 223). When the concentration of **Z-6** is increased from 0.5 to 20 μM, the CO yield and TON for C-1 can increase to nearly 400 μmol and 2000, indicating a significantly enhanced efficiency for light energy conversion (Fig. 3C and Table S1). Remarkably, the TON can reach as high as 20 403 with respect to **Z-6** under the optimized conditions, representing the highest value among the homogeneous noble-metal-free catalytic systems (Table S2). Moreover, nearly 100% selectivity for CO was achieved in these catalytic systems. The calculated quantum yields (QY) for CO generation were determined to be 21.7% for **Z-4** and 29.7% for **Z-6** upon light excitation with 488 nm. More impressively, the catalytic activity of **Z-6** was significantly higher than that of the typical noble-metal PSs (**Ru-1** and **Ir-1**) under the same conditions, signifying a great advance in the development of noble-metal-free PSs for efficient CO_2_ reduction (Table S2). Furthermore, no or trace amounts of CO evolved in the absence of PS, C-1, BIH or light, verifying that all the above factors are essential for efficient conversion of CO_2_ to CO (Fig. S30B and Table S3). To further explore the origin of CO, the catalytic experiment was performed under Ar. As shown in Fig. S30B, no CO was detected by replacing CO_2_ with Ar, which preliminarily confirmed that the CO was derived from CO_2_ photoreduction. This result was further supported by the isotope-labeling experiment under ^13^CO_2_, in which ^13^CO was detected by using gas chromatography-mass spectrometry analysis (Fig. S30C).

According to these systematic catalytic results, the structure–activity relationship of these Zn PSs can be concluded as follows. The Zn complex with anthracyl (**Z-2**) exhibited significantly higher catalytic activity than that with phenyl (**Z-1**). The catalytic activity of **Z-2** can be further improved by introducing hydrophilic PEG (**Z-4** and **Z-6**). In these PSs, the **Z-2**/**Z-4**/**Z-6** can maintain excellent orthogonal configuration with the assistance of dimethyl. By removing the methyl groups, the catalytic systems with Zn PSs (**Z-3** and **Z-5**) exhibit poor photocatalytic stability and much lower performance for CO_2_ photoreduction. To rationalize these structure–activity relationships, comprehensive investigations were carried out by using steady/transient spectra, electrochemistry and theoretical calculations.

### Steady and transient spectra

**Figure 4.**
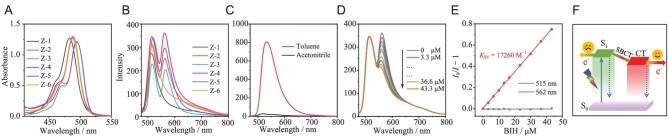
Steady spectra. (A) UV–vis absorption spectra and (B) FL spectra of Zn PSs in acetonitrile. (C) FL spectra of **Z-4** in toluene and acetonitrile. (D) FL quenching of **Z-4** with BIH as the quencher in acetonitrile. (E) Stern–Volmer plot of **Z-4**. (F) Photophysical processes of Zn PSs upon visible-light excitation. S_0_ and S_1_ represent the ground state and singlet excited state; CT is charge-transfer state. FL spectra of Zn PSs were performed under Ar. *λ*_ex_ = 455 nm, *c*_PS_ = 10.0 μM.

As shown in Fig. 4A and Fig. S31, all these Zn PSs exhibited a strong absorption peak at ∼500 nm with a molar extinction coefficient of >120 000 M^−1^ cm^−1^, indicating their strong visible-light-absorbing ability with great potential for the utilization of solar energy. Moreover, the absorption peak of **Z-3**/**Z-5** at 495 nm exhibited a 10-nm redshift as compared with that of **Z-2**/**Z-4**/**Z-6** at 488 nm. These results suggest that **Z-2**/**Z-4**/**Z-6** can maintain excellent orthogonal configuration with the assistance of dimethyl, resulting in a weak electronic interaction between the anthryl and the dipyrrin in these complexes. In addition, a triple absorption peak at ∼370 nm was observed for **Z-2**−**Z-6**, which could be ascribed to the π–π* transition of anthryl (Fig. 4A).

The excited state properties of these PSs were investigated by using photoluminescence (PL) spectra and time-resolved emission spectra (Fig. 4 and Figs S32–S34). All these complexes (**Z-1**−**Z-6**) exhibited an emission peak at ∼515 nm, which could be attributed to locally excited (^1^LE) state emission (Fig. 4B and Fig. S32). Their fluorescence QY were <0.1% in acetonitrile, providing the possibility for efficient intramolecular CT (SI Appendix, Table S4). Otherwise, a new peak at ∼563 nm was observed in degassed acetonitrile for PSs **Z-2**−**Z-5** (Fig. 4B and Fig. S32). Notably, the emission intensity at 515 nm was dramatically enhanced in toluene and the peak at ∼563 nm disappeared (Fig. 4C) under the same measurement conditions, indicating that they were sensitive to the solvent polarity and conformed to the feature of the CT state. The emission at 563 nm of Zn PSs could be attributed to the singlet CT state (^1^CT) due to their short-lived feature (<3 ns). The shoulder peak at ∼563 nm under Ar significantly decreased after exposing this solution to air, signifying an intermolecular electron/energy transfer process from the CT state PS to the oxygen molecules (Fig. S32). Additionally, owing to its short excited state lifetime and low triplet quantum yield, the excitation energy of **Z-1** was mainly consumed and inactivated by nonradiative transitions, which can rationalize the absence of CT emissions at ∼563 nm. **Z-6** exhibited poor fluorescence quantum yield (< 0.1%), high triplet state quantum yield (54%) and high performance for CO_2_ reduction. Accordingly, the presence of an efficient CT process in **Z-6** can be proposed; however, it presents a style of ‘dark state’ due to its CT character [56], which will be further rationalized by using femtosecond transient absorption spectra and theory calculations in the following parts.

As the concentration of BIH was >1000 times higher than that of C-1, a reductive mechanism was preliminarily proposed in these catalytic systems with Zn PSs. Under such circumstances, the initial step of electron transfer should proceed from the BIH to the excited Zn PSs to afford the reduced state. To track this process, fluorescence (FL) quenching experiments of Zn complexes by the BIH were performed to evaluate the intermolecular electron-transfer efficiency. The intensity of the PL peak at ∼515 nm showed almost no change when the concentration of the BIH or C-1 was increased, indicating that no electron-transfer process proceeded between singlet excited state of Zn PSs and BIH (Fig. 4D and E, and Fig. S34). Notably, the peak at ∼563 nm can be efficiently quenched by BIH with a quenching constant of >17 260 M^−1^, indicative of an efficient electron transfer from BIH to CT states of these Zn PSs (Table S5). These results preliminarily confirmed that CT states of Zn PSs can trigger intermolecular electron transfer, which was further supported by using cyclic voltammetry (CV) and transient absorption spectra (Fig. 4F).

### Electrochemistry of Zn–dipyrrin PSs

**Figure 5.**
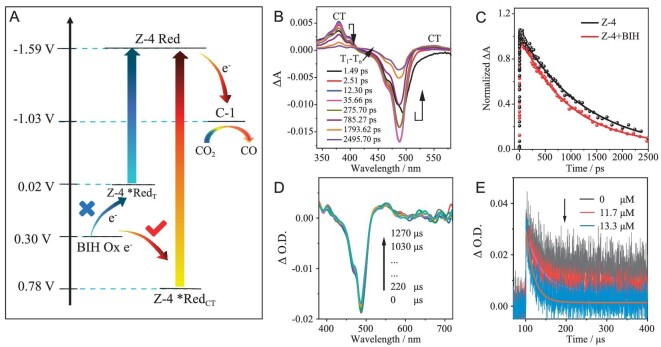
Electrochemistry and femtosecond/nanosecond transient absorption spectra. (A) Potential energy diagrams of the photocatalytic system with **Z-4** for CO_2_ photoreduction (V vs. SCE). Femtosecond transient absorption spectra of (B) **Z-4** and (C) kinetic traces of **Z-4** before and after adding BIH (10 mM) followed at 379 nm. Nanosecond transient absorption spectra of (D) **Z-4** in the presence of BIH (10 mM). (E) Kinetic traces of reduced **Z-4** with different concentrations of C-1 followed at 490 nm. These spectra were recorded in deaerated acetonitrile upon pulsed excitation at 500 nm under Ar, c_Z__–__4_ = 10 μM.

The thermodynamic feasibility of intramolecular electron transfer in these Zn PS molecules and intermolecular transfer in the catalytic systems were investigated by using CV (Fig. 5A, Fig. S35, Table 1 and Tables S6 and S7). As shown in Table 1, the reduction and oxidation potentials of PSs **Z-1**−**Z-6** were estimated to be in the ranges of −1.45 to −1.62 V and 0.75–0.89 V (vs. saturated calomel electrode (SCE)), respectively, which were further used for calculating their excited state redox potentials. In addition, their electrochemical gaps (Δ*E*_red_) and optical ^1^LE gap (*E*^S^_00_) were determined to be between 2.30/2.37 and 2.46/2.51 V in acetonitrile, respectively, manifesting that the value of Δ*E*_red_ is smaller than that of *E*^S^_00_. This result supported that the intramolecular CT processes are thermodynamic feasible for **Z-1**−**Z-6**. This viewpoint was further supported by the negative Gibbs free energy changes of the intramolecular CT process (ΔG_cs_) for all PSs.

**Table 1.** Electrochemical and optical properties of Zn–dipyrrin complexes.^a^


PSs	*E*_ox_/V	*E*_red1_/V	*E*_red2_/V	Δ*E*_red_^b^/V	*E* ^S^_00_^c^/eV	Δ*E*_red_ – *E*_00_	Δ*G*_cs_^d^/eV

**Z-1**	0.75	−1.62	−1.84	2.37	2.51	−0.14	−0.63
**Z-2**	0.77	−1.58	−1.81	2.35	2.48	−0.13	−0.62
**Z-3**	0.83	−1.47	−1.73	2.30	2.46	−0.16	−0.65
**Z-4**	0.78	−1.59	−1.84	2.37	2.48	−0.11	−0.60
**Z-5**	0.79	−1.45	−1.67	2.33	2.46	−0.13	−0.61
**Z-6**	0.81	−1.56	−1.80	2.37	2.48	−0.11	−0.60

a The experiments were carried out in deaerated acetonitrile solution containing 0.5 mM of PS with ferrocene (Fc) and 0.1 M of Bu_4_NPF_6_. Fc was used as an internal reference (*E*_1/2_ = +0.4 V (Fc^+^/Fc) vs. SCE). Glassy carbon electrode, Ag/AgNO_3_ and Pt silk were used as the working electrode, reference electrode and counter electrode, respectively. Scan rate: 0.05 V/s. ^b^Electrochemical gap determined by the energy difference between the first oxidation potential and the first reduction potential. ^c^The optical ^1^LE gap is determined by the cross point of the UV–vis absorption and fluorescence emission spectra of Zn PSs in acetonitrile. ^d^The Gibbs free energy changes of the SBCT process in acetonitrile.

Furthermore, the excited state reduction potentials of these Zn–dipyrrin complexes were deduced as 0.78–0.92 V based on the CT state (*Red_CT_) and −0.01 to –0.16 V (vs. SCE) based on the triplet state (*Red_T_), respectively (Table S7). The value of *Red_CT_ was much more positive than that of the oxidation potential of BIH (0.30 V vs. SCE), confirming that electron transfer from BIH to the CT state was thermodynamically feasible. By comparison, *Red_T_ was much more negative than that of the oxidation potential of BIH, excluding the thermodynamic feasibility for electron transfer from BIH to the triplet state of Zn PSs (Fig. 5A). Moreover, their reduction potentials were more negative than that of C-1 (−1.03 V vs. SCE), indicating that the electron transfers from the reduced Zn PSs to C-1 were thermodynamically permissible. Based on the above analysis, electron transfer can be triggered by the CT state of Zn PSs and the whole catalytic process was dominated by the reductive mechanism. This result was highly consistent with the analysis of the FL quenching experiments, and was further supported by the transient absorption spectra.

### Femtosecond/nanosecond-resolved transient absorption spectra

Femtosecond/nanosecond transient absorption spectra of Zn(II) PSs with C-1 or BIH were conducted to unveil their intramolecular photophysical properties and track the photocatalytic processes (Fig. 5 and Figs S36–S41). Upon excitation at 500 nm, two positive absorption peaks at ∼379 and ∼550 nm emerged for **Z-4** within 13 ps, which matched well with the characteristic absorption peaks of ^1^CT state. Meanwhile, the bleaching at ∼488 nm recovered within 13 ps, indicating the occurrence of a fast intramolecular CT process (Fig. 5B). Afterwards, a slow charge recombination was observed within 1.3 ns. After the addition of BIH, the lifetime of the CT state reduced from 1.3 ns to 960.3 ps (Fig. 5C) due to the electron transfer from BIH to ^1^CT state (Table S8).

**Figure 6.**
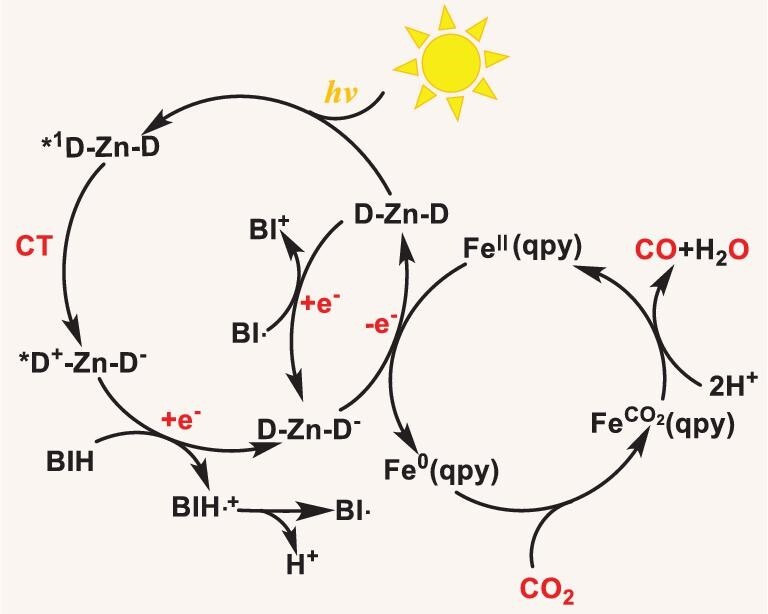
Photocatalytic process. Proposed photochemical process for CO_2_ reduction with **Z-4** (D–Zn–D). Zn is the coordination center; D is the dipyrrin ligand.

Nanosecond transient absorption spectra of Zn(II) PSs with C-1 or BIH were conducted to unveil their triplet state properties and track the photocatalytic processes (Figs S37–S41). Two positive absorption bands between 380/450 and 530/700 nm, and a strong bleaching at ∼485 nm were observed for **Z-4** (Fig. S39). This bleaching band matched well with its steady absorption, indicating the triplet state population on the dipyrrin ligand. Notably, the bleaching band for **Z-4** with BIH did not show significant decay, with >1600 μs in nanosecond transient absorption spectra (Fig. 5D), manifesting the formation of a long-lived new species, whose lifetime was much longer than that of its triplet state (130.3 μs). Subsequently, we proposed that an efficient electron transfer occurs from BIH to ^1^CT state of **Z-4** to produce a long-lived reduced state, which was also well supported by using CV and FL quenching experiments (Figs 4 and 5A). The lifetime of reduced **Z-4** becomes dramatically shorter with increasing concentrations of C-1 (Fig. 5E), indicating an efficient electron transfer from the reduced **Z-4** to C-1. As a result, the electron-transfer process for the catalytic system with **Z-4** was determined to be a reductive mechanism. As shown in Fig. 6, upon visible-light irradiation, the singlet excited state of **Z-4** can transfer electrons from one excited dipyrrin ligand to another via intramolecular electron transfer to achieve the CT state, which can further reach the long-lived triplet state of **Z-4** by ISC (intersystem crossing). Subsequently, its CT state can accept electrons from BIH to afford the long-lived reduced state, which can further deliver the electrons to C-1 for CO_2_ reduction. These consecutive and efficient intra-/intermolecular electron-transfer processes greatly boost CO_2_ reduction. In addition, the catalytic system of the left Zn–dipyrrin complexes also exhibit similar electron-transfer processes to that of **Z-4** (Figs S36–S41).

According to the experimental results, the intra-/intermolecular electron transfers during the photocatalysis were comprehensively uncovered for deciphering the structure–activity relationship. After replacing phenyl with anthryl, **Z-2** exhibited a significantly higher sensitizing ability than that of **Z-1**. This could be attributed to the lower excitation energy loss of **Z-2** than that of **Z-1** owing to a larger torsional resistance between anthryl/dipyrrin than that between phenyl/dipyrrin (Table S4). Upon visible-light excitation, CT states of these Zn PSs can be achieved by an intramolecular CT process to accept electrons from BIH to afford the long-lived reduced PS, which can trigger the intermolecular electron transfer to efficiently sensitize C-1 for boosting CO_2_-to-CO conversion. Compared with **Z-2**, the solubility of both **Z-4** and **Z-6** was significantly enhanced by the introduction of PEG, which contributed to fully exposing their active sites to improve their catalytic performance. Remarkably, the catalytic system with **Z-6** and C-1 exhibits outstanding performance with >20 000 TON, 29.7% quantum yield and 100% selectivity, which was much superior to that of noble-metal PSs **Ir-1** and **Ru-1**. However, after removing the methyl groups from **Z-4** and **Z-6**, the resulting **Z-3** and **Z-5** PSs exhibited a significant decrease in catalytic activity due to massive excitation energy loss with the anthracyl rotation and their poor photocatalytic stability. In a word, the well-defined structure–activity relationship of these Zn PSs contributed to broadening the design concept of efficient noble-metal-free PSs for boosting CO_2_ photoreduction.

## CONCLUSION

A library of Zn–dipyrrin complexes was designed and synthesized to couple Fe-based catalysts to construct Earth-abundant catalytic systems for CO_2_ photoreduction. The Zn–dipyrrin complexes were first explored for CO_2_ photoreduction and the influence of the CT effect on CO_2_ reduction was discovered in this work. Detailed mechanistic research demonstrated that the CT states of these Zn PSs can be achieved by an intramolecular CT process and the CT states can directly accept electrons from the BIH to form long-lived reduced PSs. The formation of the long-lived reduced PS can trigger the intermolecular electron transfer to efficiently sensitize C-1 for boosting CO_2_-to-CO conversion upon visible-light excitation, which does not require long-lived triplet states to avoid the decrease in the electrochemical driving force for the intermolecular electron transfer. Remarkably, the Earth-abundant catalytic system with **Z-6** exhibited a high catalytic performance with >20 000 TON and 29.7% quantum yield. This was highly superior to that of noble-metal PSs (**Ir-1** and **Ru-1**) and represents excellent catalytic performance among the homogeneous noble-metal-free catalytic systems. This work opens up a new avenue to boost CO_2_ photoreduction by rationally designing low-cost PSs with more abundant metals.

## METHODS

Full experimental details and procedures for the synthesis of the compounds used in the present study, molecular structures and details of **Z-1**–**Z-6** are described in the Supplementary Data.

## DATA AND SOFTWARE AVAILABILITY

All data needed to evaluate the conclusions in the paper are present in the paper and/or the Supplementary materials. Additional data related to this paper may be requested from the authors.

## Supplementary Material

nwae130_Supplemental_File
