# Supplementary material for: Earth-abundant Zn–dipyrrin chromophores for efficient CO2 photoreduction
Source: Natl Sci Rev. 2024 Apr 1;11(6):nwae130. doi: 10.1093/nsr/nwae130 (PMC11089819; doi:10.1093/nsr/nwae130)
Supplement: nwae130_Supplemental_File [file nwae130_supplemental_file.pdf]

Supplementary Information

Earth-abundant Zn-dipyrrin chromophores for efficient CO_2 photoreduction

*Song Guo, Fu-Gui Zeng, Xiao-Di Li, Kai-Kai Chen, Ping Wang, Tong-Bu Lu and Zhi-Ming Zhang**

Institute for New Energy Materials and Low Carbon Technologies, School of Materials Science & Engineering, Tianjin University of Technology, Tianjin 300384, China

*E-mail: zmzhang@email.tjut.edu.cn

Table of Contents

Experimental Section 1
Materials and methods. 1
Instruments 1
Photocatalytic CO_2 reduction 1
Quantum yield for CO generation 2
Triplet Quantum Yield 2
Gibbs free energy changes 2
Femtosecond transient absorption spectra 3
Synthetic process of Z-1 – Z-6 4
Supplementary Figure 1 4
Synthesis of 2. 5
Synthesis of Z-1[1]. 5
Synthesis of 4. 5
Synthesis of Z-2[1]. 5
Synthesis of 6. 6
Synthesis of 7. 6
Synthesis of 8. 6
Synthesis of Z-3[1]. 6
Synthesis of 10. 7
Synthesis of 11. 7
Synthesis of 12. 7
Synthesis of Z-4. 8
Synthesis of 14. 8
Synthesis of 15. 9
Synthesis of 16. 9
Synthesis of Z-6. 9
Synthesis of 18. 10
Synthesis of 19. 10
Synthesis of Z-5. 10
Structural characterization 12
Supplementary Figure 2. ^1H NMR spectrum of **Z-1** (400 MHz, $CDCl_3$). 12
Supplementary Figure 3. HRMS ESI of **Z-1** 12
Supplementary Figure 4. ^{13}C NMR spectrum of **Z-1** (100 MHz, $CDCl_3$). 13
Supplementary Figure 5. ^1H NMR spectrum of **Z-2** (400 MHz, $CDCl_3$). 13
Supplementary Figure 6. HRMS ESI of **Z-2.** 14
Supplementary Figure 7. ^{13}C NMR spectrum of **Z-2** (100 MHz, $CDCl_3$) 14
Supplementary Figure 8. ^1H NMR spectrum of **6** (400 MHz, $CDCl_3$) 15
Supplementary Figure 9. ^1H NMR spectrum of **7** (400 MHz, $CDCl_3$). 15
Supplementary Figure 10. ^1H NMR spectrum of **Z-3** (400 MHz, $CDCl_3$) 16
Supplementary Figure 11. HRMS ESI of **Z-3** 16
Supplementary Figure 12. ^{13}C NMR spectrum of **Z-3** (100 MHz, $CDCl_3$) 17
Supplementary Figure 13. ^1H NMR spectrum of **10** (400 MHz, $CDCl_3$) 17
Supplementary Figure 14. ^1H NMR spectrum of **11** (400 MHz, $CDCl_3$). 18
Supplementary Figure 15. ^1H NMR spectrum of **12** (400 MHz, $CDCl_3$). 18

Supplementary Figure 16. ^{1}H NMR spectrum of **Z-4** (400 MHz, $CDCl_3$)....19
Supplementary Figure 17. HRMS ESI of **Z-4**....19
Supplementary Figure 18. ^{13}C NMR spectrum of **Z-4** (400 MHz, $CDCl_3$)....20
Supplementary Figure 19. ^{1}H NMR spectrum of **14** (400 MHz, $CDCl_3$)....20
Supplementary Figure 20. ^{1}H NMR spectrum of **15** (400 MHz, $CDCl_3$)....21
Supplementary Figure 21. ^{1}H NMR spectrum of **16** (400 MHz, $CDCl_3$)....21
Supplementary Figure 22. ^{1}H NMR spectrum of **Z-6** (400 MHz, $CDCl_3$)....22
Supplementary Figure 23. HRMS ESI of **Z-6**....22
Supplementary Figure 24. ^{13}C NMR spectrum of **Z-6** (400 MHz, $CDCl_3$)....23
Supplementary Figure 25. ^{1}H NMR spectrum of **18** (400 MHz, $CDCl_3$)....23
Supplementary Figure 26. ^{1}H NMR spectrum of **19** (400 MHz, $CDCl_3$)....24
Supplementary Figure 27. ^{1}H NMR spectrum of **Z-5** (400 MHz, $CDCl_3$)....24
Supplementary Figure 28. HRMS ESI of **Z-5**....25
Supplementary Figure 29. ^{13}C NMR spectrum of **Z-5** (400 MHz, $CDCl_3$)....25
Supplementary Figures....26
Supplementary Figure 30....26
Supplementary Figure 31....27
Supplementary Figure 32....27
Supplementary Figure 33....28
Supplementary Figure 34....28
Supplementary Figure 35....29
Supplementary Figure 36....29
Supplementary Figure 37....30
Supplementary Figure 38....31
Supplementary Figure 39....32
Supplementary Figure 40....33
Supplementary Figure 41....34
Supplementary Tables....35
Supplementary Table 1....35
Supplementary Table 2....35
Supplementary Table 3....36
Supplementary Table 4....36
Supplementary Table 5....36
Supplementary Table 6....37
Supplementary Table 7....37
Supplementary Table 8....37
References....38

Experimental Section

Materials and methods.

All the chemicals for the synthesis are analytical pure, which were used without further purification. Solvents were dried and distilled before use. 2-methylpyrrole (Purity: 97.0%), DDQ (Purity: 98.0%) and *p*-chloranil (Purity: 98.0%) were purchased from HEOWNS. 2,4-dimethylpyrrole (Purity: 97.0%) and NH_4PF_6 were bought from Alfa Aesar. $Pd(PPh_3)_4$ was purchased from Sigma-Aldrich. Chromatographic-grade dichloromethane and methanol were bought from Adamas. The synthetic scheme of Zn-dipyrrin complexes (**Z-1** − **Z-6**) is presented in Supporting Information, Fig. S1. The intermediates and target complexes were characterized by NMR and HRMS.

Instruments.

^{1}H NMR spectra were recorded with an AVANCE III HD 400 MHz. ^{13}C NMR spectra were performed on an AVANCE III HD 100 MHz with total proton decoupling. High resolution mass spectra (HRMS) were detected on Q-TOF LC-MS with an ESI mode. UV-vis absorption spectra were recorded on a LAMBDA750 spectrophotometer. FL spectra were taken on Hitachi F4600 spectrofluorometer. FL lifetime was carried out by transient state fluorescence spectrometer (Edinburgh FLS1000). Transient absorption spectra were measured on the LP980 laser flash photolysis instrument (Edinburgh, UK). Electrochemical measurements were measured on a CHI 760E electrochemical workstation at room temperature. 300 W Xenon lamp (CEL-HXF300, CEAULICHT) with 420 nm filter was employed as a visible light source for photocatalytic CO_2 reduction. Photoluminescence quantum yields of PSs were performed by FLS1000 Photoluminescence Spectrometer with integral sphere (Edinburgh, UK).

Photocatalytic CO_2 reduction.

Photocatalytic conversion of CO_2 to CO was conducted in a reactor (total volume of 105.5 mL) containing PS (0.5 μM), C-1 (10.0 μM), BIH (20.0 mM) and H_2O (0.8 mM) in 20.0 mL CH_3CN. After bubbled with CO_2 for 30 min, the reaction mixture was irradiated with a 300 W xenon lamp (λ > 420 nm, 80 mW•cm^{-2}) at 25 °C. The generated gases were analyzed by gas chromatography (SHIMADZU GC-2014), equipped with Porapak N (GS-Tek) and MOLSIEVE 13X (Agilent) packed columns, with a thermal conductivity detector (TCD) for H_2 determination, and flame ionization detector (FID) for CO determination. The ^{13}C-labeled experiments were conducted under $^{13}CO_2$ atmosphere and the gaseous products analyzed by a gas chromatograph mass spectrometer GC-MS (HIDEN ANALYTICAL, HPR20).

Quantum yield for CO generation.

The incident light intensity was estimated as 2.09 × 10^{-9} einstein s^{-1} by ferrioxalate actinometry using 480 nm monochhromator. Reaction condition: PS (5.0 μM), C-1 (10.0 μM), BIH (15.0 mM), H_2O (3.3 mM), in 5 mL CH_3CN. The gaseous products were analysed by GC-FID. The quantum yield for CO_2-to-CO conversion after irradiation for 10 h was calculated using the following equation:

$$\text{Quantum Yield}(\Phi) = \frac{number\ of\ CO\ molecules}{number\ of\ incident\ photons} \times 100\%$$

Triplet quantum yield.

The triplet quantum yield was measured by ground state depletion method. The absorbance of the samples or the reference compounds are adjusted to about 0.3 at the excitation wavelength to avoid multiphoton and TTA effect. The using of same excitation wavelength and optically matched solutions are to make sure the same amount of photons were absorbed by the sample and the reference compounds. The decay trace of bleaching band was monitored and the O.D. value was obtained. The data fitting was performed by the software of LP980 laser flash photolysis spectrometer (Edinburgh Instruments, UK).

$$\boldsymbol{\Phi}_{\mathbf{T}}^{\mathbf{Sam}} = \boldsymbol{\Phi}_{\mathbf{T}}^{\mathbf{Ref}} \times \frac{\Delta \boldsymbol{A}_{\mathbf{Sam}}}{\Delta \boldsymbol{A}_{\mathbf{Ref}}} \times \frac{\boldsymbol{\varepsilon}_{\boldsymbol{S}}^{\boldsymbol{Ref}}}{\boldsymbol{\varepsilon}_{\boldsymbol{S}}}$$

Gibbs free energy changes

The Gibbs free energy changes for the SBCT process was estimated by eq 1 and 2.

$$\Delta G^{0}{}_{\mathrm{CS}} = e[E_{OX} - E_{RED}] - E_{00} + \Delta G_{\mathrm{S}} \qquad \text{<eq 1.>}$$

$$\Delta G_{\mathrm{S}} = -\frac{e^2}{4\pi\varepsilon_{\mathrm{S}}\varepsilon_0 R_{\mathrm{CC}}} - \frac{e^2}{8\pi\varepsilon_0}\left(\frac{1}{R_D} + \frac{1}{R_{\mathrm{A}}}\right)\left(\frac{1}{\varepsilon_{REF}} - \frac{1}{\varepsilon_S}\right) \qquad \text{<eq 2.>}$$

where e is the electronic charge, E_{OX} is the oxidation potential of electron-donor unit, E_{RED} is the reduction potential of the electron-acceptor unit, E_{00} is the approximate energy level obtained from the onset of fluoresce emission (5% relative intensity). ΔG_{S} is the static columbic energy, which was estimated from eq 2. ε_{S} is the static dielectric constant of the solvent, R_{CC} is the center-to-center separation distance between the electron donor (dipyrrin) and electron acceptor (dipyrrin). R_{D} is the radius of the electron donor, R_{A} is the radius of the electron acceptor, $\varepsilon_{\mathrm{REF}}$ is the static dielectric constant of the solvent used for electrochemical study, and ε_0 is the permittivity of free space.

Femtosecond transient absorption spectra.

The femtosecond pump-probe TA measurements were performed using a regenerative amplified Ti: sapphire laser system (Coherent; 800 nm, 70 fs, 6 mJ/pulse, and 1 kHz repetition rate) as the laser source and a femto-TA 100 spectrometer (Time-Tech Spectra). Briefly, the 800 nm output pulse from the regenerative amplifier was split in two parts with a 50% beam splitter. The transmitted part was used to pump a TOPAS Optical Parametric Amplifiers (OPA) which generated a wavelength-tunable laser from 250 nm to 2.5 mm as pump beam. The reflected 800 nm beam was split again into two parts. One part with less than 10% was attenuated with a neutral density filter and focused into a 2 mm thick sapphire window to generate a white light continuum (WLC) used for probe beam. The probe was focused with an Al parabolic reflector onto the sample. After the sample, the probe beam was collimated and then focused into a fiber-coupled spectrometer with CMOS sensors and detected at a frequency of 1 kHz. The intensity of the pump pulse used in the experiment was controlled by a variable neutral-density filter wheel. The delay between the pump and probe pulses was controlled by a motorized delay stage. The pump pulses were chopped by a synchronized chopper at 500 Hz and the absorbance change was calculated with two adjacent probe pulses (pump-blocked and pump-unblocked). The samples were placed in 2 mm cuvettes and were vigorously stirred in all measurements.

Synthetic process of Z-1 – Z-6

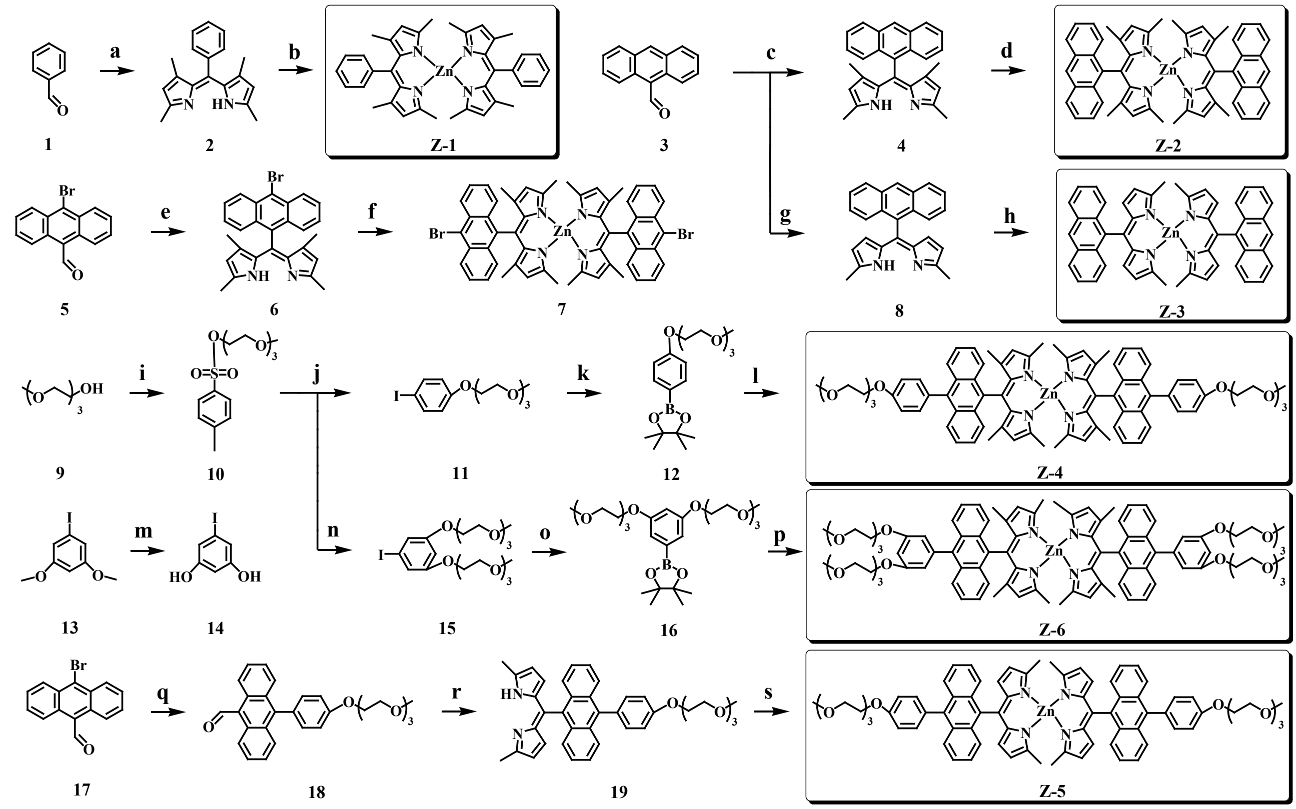

Supplementary Figure 1. Synthetic route of **Z-1** – **Z-6**. (a) 2,4-dimethylpyrrole, CH_2Cl_2, TFA, DDQ, Ar, yield: 32.1%. (b) CH_2Cl_2, zinc acetate, MeOH, Ar, yield: 81.1%. (c) 2,4-dimethylpyrrole, CH_2Cl_2, TFA, *p*-chloranil, Ar, yield: 84.5%. (d) CH_2Cl_2, zinc acetate, MeOH, Ar, yield: 94.9%. (e) 2,4-dimethylpyrrole, CH_2Cl_2, TFA, *p*-chloranil, Ar, yield: 79.5%. (f) CH_2Cl_2, zinc acetate, MeOH, Ar, yield: 94.5%. (g) 2-methylpyrrole, CH_2Cl_2, TFA, *p*-chloranil, Ar, yield: 79.5%. (h) CH_2Cl_2, zinc acetate, MeOH, Ar, yield: 90.9%. (i) TsCl, pyridine, 0 °C for 2 h and rt for 18 h, yield: 82.0%. (j) 4-iodophenol, K_2CO_3, CH_3CN, refluxed for 24 h, yield: 78.5%. (k) bis(pinacolato)diboron, potassium acetate, DMSO, $PdCl_2(dppf)_2$, 90 °C under Ar for 14 h, yield: 95.1%. (l) compound 7, Na_2CO_3, $Pd(PPh_3)_4$, toluene/ethanol/water, 90 °C, 6 h, under Ar, yield: 59.6%. (m) CH_2Cl_2, BBr_3, 1-iodo-3,5-dimethoxybenzene, -78°C, Ar, yield: 65.5%. (n) 5-iodobenzene-1,3-diol, K_2CO_3, CH_3CN, refluxed for 24 h, yield: 65.5%. (o) bis(pinacolato)diboron, potassium acetate, DMSO, $PdCl_2(dppf)_2$, 90 °C under Ar for 14 h, yield: 35.5%. (p) compound 7, Na_2CO_3, $Pd(PPh_3)_4$, toluene/ethanol/water, 90 °C, 12 h, under Ar, yield: 39.5%. (q) compound 12, Na_2CO_3, $Pd(PPh_3)_4$, toluene/ethanol/water, 90 °C, 12 h, under Ar, yield: 60.1%. (r) 2-methylpyrrole, CH_2Cl_2, TFA, *p*-chloranil, Ar, yield: 84.5%. (s) CH_2Cl_2, zinc acetate, MeOH, Ar, yield: 91.1%.

Synthesis of 2.

Benzaldehyde (**1**) (764.1 mg, 7.2 mmol) and 2,4-dimethylpyrrole (1.8 mL, 17.8 mmol) were dissolved in dry dichloromethane (100.0 mL) under Ar. Then trifluoroacetic acid (30 μL) was added into the mixture under the ice bath condition. After stirred at room temperature for 12 h, DDQ (1.7 g, 7.5 mmol) in dried dichloromethane (20.0 mL) was added to above reaction system and the mixture was kept for another 2 h at room temperature. Next, the reaction solution was concentrated under reduced pressure to afford the sticky liquid, which was further washed with water (100.0 mL). Then the solution was extracted with CH_2Cl_2 and the organic layer was collected and dried over anhydrous $MgSO_4$. The solvent was evaporated under reduced pressure to give crude product, which was further purified by alumina column chromatography (dichloromethane/methanol = 30/1, v/v) to give **2** as brown solid. Yield: 443.1 mg (32.1%).

Synthesis of Z-1[1].

2 (276.1 mg, 1.0 mmol) in dichloromethane (20.0 mL) was mixed with zinc acetate (110.4 mg, 0.5 mmol) in methanol (10.0 mL). After stirring for overnight, the reaction solution was reduced by a rotary evaporator to afford the brown precipitate, which was recrystallized, filtered and rinsed with cold methanol. Then the crude product was further purified by silica gel column chromatography (dichloromethane/ n-hexane = 2/1, v/v) to give **Z-1** as brown solid. Yield: 248.4 mg (81.1%). ^{1}H NMR (400 MHz, $CDCl_3$) δ (ppm) = 7.44–7.41 (m, 6H), 7.33 – 7.31 (m, 4H), 5.89 (s, 2H), 2.35 (s, 6H), 1.29 (s, 6H). ^{13}C NMR (100 MHz, $CDCl_3$) δ = 156.77, 144.58, 144.22, 140.06, 135.59, 129.37, 128.61, 127.96, 120.26, 16.21, 15.64. ESI-HRMS: $C_{38}H_{38}N_4Zn$ = 616.1270 (calc.); m/z = 616.2460 (found).

Synthesis of 4.

9-anthracenecarboxaldehyde (**3**) (206.1 mg, 1.0 mmol) in dichloromethane solution (30.0 mL) were mixed with 2,4-dimethylpyrrole (225.0 μL, 2.3 mmol) and trifluoroacetic acid (30.0 μL) under Ar atmosphere. After stirring for overnight, *p*-chloranil (245.4 mg, 1.0 mmol) was added to the above solution. After stirred for another 2 h, the solvent was removed by rotary evaporator and the residue was purified by alumina column chromatography (hexane/dichloromethane = 2/1). The orange-brown band was collected and evaporated to give the product as yellow powder. Yield: 318.4 mg (84.5%).

Synthesis of Z-2[1].

4 (376.1 mg, 1.0 mmol) in dichloromethane (20.0 mL) was mixed with zinc acetate (110.4 mg, 0.5 mmol) in methanol (10.0 mL). After stirring overnight, the reaction solution was reduced by a rotary evaporator to afford the brown precipitate, which was recrystallized, filtered and rinsed with cold methanol. Then the

crude product was further purified by silica gel column chromatography (dichloromethane/ n-hexane = 2/1, v/v) to give **Z-2** as orange-brown solid. Yield: 385 mg (94.9%). ^{1}H NMR (400 MHz, $CDCl_3$) δ (ppm) = 8.57 (s, 2H), 8.05 – 8.00 (m, 8H), 7.47 – 7.39 (m, 4H), 7.37–7.36 (m, 4H), 5.90 (s, 4H), 2.34 (s, 12H), 0.48 (s, 12H). ^{13}C NMR (100 MHz, $CDCl_3$) δ = 156.79, 143.89, 140.78, 136.50, 134.02, 131.60, 130.87, 128.14, 127.22, 126.31, 125.61, 125.39, 120.31, 16.59, 14.95. ESI-HRMS: $C_{54}H_{46}N_4Zn$ = 816.3788 (calc.); *m/z* = 816.3217 (found).

Synthesis of 6.

Compound **5** (285.0 mg, 1.0 mmol) in dichloromethane (30.0 mL) was mixed with 2,4-dimethylpyrrole (225.0 μL, 2.3 mmol) and trifluoroacetic acid (30.0 μL) under Ar atmosphere. After stirring overnight, *p*-chloranil (245.4 mg, 1.0 mmol) was added to the solution. After stirred for another 2 h, the solvent was removed by rotary evaporator and the residue was purified by alumina column chromatography (hexane/dichloromethane = 2/1). The orange-brown band was collected and evaporated to give the product. Yield: 362.1 mg (79.5%). ^{1}H NMR (400 MHz, $CDCl_3$) δ = 8.56 (d, *J* = 8.9 Hz, 2H), 8.04 (d, *J* = 8.7 Hz, 2H), 7. 62–7.56 (m, 2H), 7.44-7.38 (m, 2H), 5.77 (s, 2H), 2.41 (s, 6H), 0.51 (s, 6H).

Synthesis of 7.

6 (455.4 mg, 1.0 mmol) in dichloromethane (20.0 mL) was mixed with zinc acetate (110.4 mg, 0.5 mmol) in methanol (10.0 mL). After stirring for overnight, the reaction solution was reduced by a rotary evaporator to afford the brown precipitate, which was recrystallized, filtered and rinsed with cold methanol. Then the crude product was further purified by silica gel column chromatography (dichloromethane/ n-hexane = 2/1, v/v) to give **7** as brown solid. Yield: 463.1 mg (94.5%). ^{1}H NMR (400 MHz, $CDCl_3$) δ (ppm) = 8.60 (d, *J* = 8.9 Hz, 4H), 8.06 (d, *J* = 8.7 Hz, 4H), 7.63 – 7.59 (m, 4H), 7.45 – 7.40 (m, 4H), 5.90 (s, 4H), 2.33 (s, 12H), 0.48 (s, 12H).

Synthesis of 8.

9-anthracenecarboxaldehyde (**3**) (206.1 mg, 1.0 mmol) in dichloromethane (30.0 mL) was mixed wiith 2-methylpyrrole (200.0 μL, 2.3 mmol) and trifluoroacetic acid (30.0 μL) under Ar atmosphere. After stirring overnight, *p*-chloranil (245.4 mg, 1.0 mmol) was added to the above solution. After stirred for another 2 h, the solvent was evaporated and the residue was purified by alumina column chromatography (hexane/dichloromethane = 2/1). The orange-brown band was collected and evaporated to give yellow powder. Yield: 277.6 mg (79.6%).

Synthesis of Z-3[1].

Compound **8** (455.4 mg, 1.0 mmol) in dichloromethane (20.0 mL) was mixed with zinc acetate (110.4 mg, 0.5 mmol) in methanol (10.0 mL). After stirring for overnight, the reaction solution was reduced by a rotary evaporator to afford the brown precipitate, which was recrystallized, filtered and rinsed with cold methanol. Then the crude product was further purified by silica gel column chromatography (dichloromethane/ n-hexane = 2/1, v/v) to give **Z-3** as orange-brown solid. Yield: 463.5 mg (90.9%). ^{1}H NMR (400 MHz, $CDCl_3$) δ (ppm) = 8.56 (s, 2H), 8.05 (d, *J* = 8.5 Hz, 4H), 7.97 (d, *J* = 8.7 Hz, 4H), 7.47 – 7.43 (m, 4H), 7.39 – 7.34 (m, 4H), 6.09 (s, 8H), 2.39 (s, 12H). ESI-HRMS: $C_{54}H_{38}N_4Zn$ = 760.2725 (calc.); *m/z* = 760.2440 (found). ^{13}C NMR (100 MHz, $CDCl_3$) δ =159.15, 141.01, 140.27, 133.09, 132.91, 131.41, 130.89, 128.16, 127.26, 127.00, 125.87, 125.14, 117.68, 16.82.

Synthesis of 10.

Tosyl chloride (9.3 g, 48.7 mmol) was added dropwisely to **9** (5.0 g, 30.5 mmol) in pyridine (30.0 mL). After stirring for 2 h at 0 °C, the resulting mixture was stirred for 18 h at room temperature. The reaction was carefully quenched at 0 °C by addition of 10% aqueous HCl. The organic phase was decanted, washed with water (3 × 100 mL), dried over Na_2SO_4, and concentrated under reduced pressure. Then the crude product was further purified by silica gel column chromatography (hexane/EtOAc = 10/1, v/v) to give **10** as orange-brown solid. Yield: 8.3 g (82.0%). ^{1}H NMR (400 MHz, $CDCl_3$) δ = 7.80 (d, *J* = 8.2 Hz, 2H), 7.35 (d, *J* = 8.1 Hz, 2H), 4.16 (t, *J* = 8.1 Hz, 2H), 3.70 – 3.67 (m, 4H), 3.63 – 3.52 (m, 6H), 3.37 (s, 3H), 2.45 (s, 3H).

Synthesis of 11.

4-iodophenol (3.3 g, 16.7 mmol) and K_2CO_3 (3.2 g, 23.4 mmol) were dissolved in dry acetonitrile (50.0 mL). Then Compound **7** (5.2 g, 15.6 mmol) was dissolved in dry acetonitrile (20.0 mL) and added slowly to the above mixture solution by a dropping funnel. The resulting solution was refluxed for 24 h and then quenched with ice water (30.0 mL). The combined organic layers were washed with water (100.0 mL), dried over Na_2SO_4 and concentrated under reduced pressure. The crude product was purified by silica gel column chromatography (dichloromethane/methanol = 50/1, v/v), affording **11** as light yellow liquid. Yield: 4.5 g (78.5%). ^{1}H NMR (400 MHz, $CDCl_3$) δ = 7.53 (d, *J* = 8.8 Hz, 2H), 6.69 (d, *J* = 8.7 Hz, 2H), 4.11 – 4.04 (m, 2H), 3.86 – 3.80 (m, 2H), 3.75 – 3.70 (m, 2H), 3.69 – 3.62 (m, 4H), 3.56 – 3.52 (m, 2H), 3.37 (s, 3H).

Synthesis of 12.

11 (6.0 g, 16.4 mmol), bis(pinacolato)diboron (7.0 g, 27.6 mmol) and potassium acetate (4.9 g, 49.1 mmol) were dissolved in DMSO (30.0 mL). After adding $PdCl_2(dppf)_2$ (1.2 g, 1.6 mmol), the reaction solution was bubbled under Ar for 15 min. The mixture was heated to 90 °C and reacted under Ar for 14 h. After cooling to room temperature, the reaction solution was poured into water and extracted with DCM. The organic phase concentrated under reduced pressure to give crude product, which was further purified by silica gel column chromatography (EtOAc/dichloromethane = 3/7, v/v) to give **12** as a pale yellow oil. Yield: 5.7 g (95.1%). ^{1}H NMR (400 MHz, $CDCl_3$) δ = 7.73 (d, J = 8.3 Hz, 2H), 6.90 (d, J = 8.2 Hz, 2H), 4.15 (t, J = 8.2 Hz, 2H), 3.85 (t, J = 8.2 Hz, 2H), 3.74 – 3.72 (m, 2H), 3.69 – 3.64 (m, 4H), 3.55 – 3.53 (m, 2H), 3.37 (s, 3H), 1.33 (s, 12H).

Synthesis of Z-4.

7 (585.0 mg, 0.6 mmol), **12** (514.0 mg, 1.2 mmol) and Na_2CO_3 (190.0 mg, 0.6 mmol) were dissolved in a mixed solvents of toluene/ethanol/water (30 mL, 2/2/1, v/v/v). After bubbling with Ar for 15min, $Pd(PPh_3)_4$ (34.7 mg, 0.03 mmol) was added into the reaction system. The resulting mixture was refluxed at 90 °C for 12 h under Ar atmosphere. After cooling down to room temperature, water (50 mL) was added to the reaction solution and the organic layer was separated from water. The obtained organic fraction was dried over Na_2SO_4 and evaporated under reduced pressure. The crude product was purified by silica gel column chromatography (dichloromethane/petroleum ether = 1/1, v/v) to afford the pure **Z-4** as brown solid. Yield: 463.7 mg (59.6%). ^{1}H NMR (400 MHz, $CDCl_3$) δ = 8.07 (d, J = 7.8 Hz, 4H), 7.72 (d, J = 8.7 Hz, 4H), 7.42 (d, J = 8.1 Hz, 4H), 7.35 (s, 8H), 7.18 (d, J = 8.2 Hz, 4H), 5.92 (s, 4H), 4.31 (t, J = 8.1 Hz, 4H), 4.01 – 3.95 (m, 4H), 3.84 (t, J = 4.1 Hz, 4H), 3.77– 3.71 (m, 8H), 3.60 (t, J = 4.1 Hz, 4H), 3.41 (s, 6H), 2.36 (s, 12H), 0.58 (s, 12H). ^{13}C NMR (100 MHz, $CDCl_3$) δ = 158.36, 156.82, 143.98, 141.25, 138.18, 136.68, 133.83, 132.61, 131.11, 130.49, 126.86, 125.95, 125.74, 125.18, 120.36, 114.51, 70.95, 70.77, 70.66, 69.89, 67.59, 59.11, 16.65, 15.04. ESI-HRMS: $C_{80}H_{82}N_4O_8Zn$ = 1292.9381 (calc.); m/z = 1292.5516 (found).

Synthesis of 14.

1-iodo-3, 5-dimethoxybenzene (**13**) (2 g, 7.6 mmol) in dry dichloromethane (60 mL) was added slowly to BBr_3 (dichloromethane 1M, 25mL, 25mol) at -78 °C and stirred for 1 h. After heated to room temperature, the mixture was stirred for 12 h. The reaction was quenched by the addition of ice water (100 mL). Then the solution was extracted with dichloromethane and the organic layer was collected and dried over anhydrous $MgSO_4$. The solvent was evaporated under reduced pressure to give crude product, which was further purified by alumina column chromatography (dichloromethane/ n-hexane = 1/1, v/v). The product was

further purified by recrystallization with dichloromethane / hexane to obtain **14** as white solid. Yield: 1.2 g (65.5%). ^{1}H NMR (400 MHz, $CDCl_3$) δ = 6.79 (d, *J* = 2.1 Hz, 2H), 6.31 (t, *J* = 2.1 Hz, 1H).

Synthesis of 15.

Compound **14** (472.0 mg, 3.0 mmol), K_2CO_3 (320.0 mg, 2.3 mmol) and **7** (500.0 mg, 1.5 mmol) were mixed in dry acetonitrile (30.0 mL). The resulting solution was refluxed for 24 h. After the reaction was completed, the mixture was quenched with ice water (30.0 mL). Then the combined organic layers were washed with water (100.0 mL), dried over Na_2SO_4 and concentrated under reduced pressure. The crude product was purified by silica gel column chromatography (dichloromethane/methanol = 40/1, v/v), affording **15** as yellow liquid. Yield: 425.6 mg (65.5%). ^{1}H NMR (400 MHz, $CDCl_3$) δ = 6.87 (s, 2H), 6.44 (s, 1H), 4.07 (t, *J* = 4.1 Hz, 4H), 3.82 (t, *J* = 4.1 Hz, 4H), 3.72 (t, *J* = 4.1 Hz, 4H), 3.69 – 3.64 (m, 8H), 3.55 (t, *J* = 4.1 Hz, 4H), 3.38 (s, 6H).

Synthesis of 16.

Compound **15** (433.2 mg, 1.0 mmol), bis(pinacolato)diboron (1.1 g, 4.0 mmol) and potassium acetate (7.0 g, 7.1 mmol) were dissolved in 30.0 mL DMSO. After bubbled with Ar for 15 min, $PdCl_2(dppf)_2$ (163.2 mg, 0.2 mmol) was added into the reaction solution. The mixture reacted at 90 ºC for 14 h with vigorous stirring under Ar. After cooling down to room temperature, the reaction solution was poured into water and extracted with DCM. The organic phase was concentrated under reduced pressure to give crude product, which was further purified by silica gel column chromatography (EtOAc/dichloromethane = 3/7, v/v) to give **16** as a pale yellow oil. Yield: 153.8 mg (35.5%). ^{1}H NMR (400 MHz, $CDCl_3$) δ = 6.95 (s, 2H), 6.60 (s, 1H), 4.13 (t, *J* = 4.4 Hz, 4H), 3.84 (t, *J* = 4.1 Hz, 4H), 3.74 – 3.72 (m, 4H), 3.69 – 3.65 (m, 8H), 3.55 (t, *J* = 4.1 Hz, 4H), 3.38 (s, 6H), 1.33 (s, 12H), 3.38 (s, 6H), 1.33 (s, 12H).

Synthesis of Z-6.

Compound **7** (585.0 mg, 0.6 mmol), Compound **16** (668.5 mg, 1.2 mmol) and Na_2CO_3 (190.0 mg, 0.6 mmol) were dissolved in toluene/ethanol/water (30.0 mL, 2/2/1, v/v/v). After bubbling with Ar for 15min, $Pd(PPh_3)_4$ (34.7 mg, 0.03 mmol) was added into the above reaction solution. The resulting mixture was refluxed at 90 °C for 12 h under Ar atmosphere. After cooling down to room temperature, water (50.0 mL) was added and the organic layer was separated from water. The obtained organic fraction was dried over Na_2SO_4 and evaporated under reduced pressure. The crude product was purified by column chromatography (dichloromethane/petroleum ether = 1/1, v/v) to afford the pure **Z-6**. Yield :387.7 mg (39.5%). ^{1}H NMR (400 MHz, $CDCl_3$) δ = 8.05 (d, *J* = 7.4 Hz, 4H), 7.75 (d, *J* = 9.7 Hz, 4H), 7.37 – 7.32 (m, 8H), 6.71 (s, 2H),

6.67 (d, J = 2.2 Hz, 4H), 5.93 (s, 4H), 4.21 (d, J = 5.1 Hz, 8H), 3.90 (t, J = 4.2 Hz, 8H), 3.78 – 3.75 (m, 8H), 3.72 – 3.65 (m, 16H), 3.57 – 3.54 (m, 8H), 3.37 (s, 12H), 2.36 (s, 12H), 0.58 (s, 12H). ^{13}C NMR (100 MHz, $CDCl_3$) δ = 159.91, 156.82, 143.97, 141.13, 140.84, 138.15, 136.65, 133.98, 130.36, 129.83, 126.83, 126.00, 125.65, 125.29, 120.38, 110.59, 71.94, 70.86, 70.67, 70.57, 69.77, 67.64, 59.03, 53.45, 16.62, 15.06. ESI-HRMS: $C_{94}H_{110}N_4O_{16}Zn$ = 1617.3054 (calc.); *m/z* = 1617.7040 (found).

Synthesis of 18.

Compound **17** (285.0 mg, 1.0 mmol), Compound **12** (546.2 mg, 1.2 mmol) and Na_2CO_3 (180 mg, 1.3 mmol) were dissolved in a mixed solvents of toluene/ethanol/water (30.0 mL, 2/2/1, v/v/v). After bubbling with Ar for 15 min, $Pd(PPh_3)_4$ (20.8 mg, 0.1 mmol) was added into the reaction system. The resulting mixture was refluxed at 90 °C for 12 h under Ar atmosphere. After cooling down to room temperature, the reaction mixture was extracted with CH_2Cl_2, washed with water (50.0 mL) and dried over Na_2SO_4. The solvent of organic phase was evaporated under reduced pressure. The crude product was further purified with silica gel column chromatography (dichloromethane/n-hexane = 1/1, v/v) to give red solid. Yield: 320.6 mg (60.1%). ^1H NMR (400 MHz, $CDCl_3$) δ = 11.58 (s, 1H), 9.01 (d, J = 9.0 Hz, 2H), 7.76 (d, J = 8.8 Hz, 2H), 7.68 – 7.63 (m, 2H), 7.44 – 7.39 (m, 2H), 7.30 (d, J = 8.6 Hz, 2H), 7.15 (d, J = 8.6 Hz, 2H), 4.39 (t, J = 8.4 Hz, 2H), 3.97 (t, J = 8.4 Hz, 2H), 3.83 – 3.79 (m, 2H), 3.76 – 3.69 (m, 4H), 3.60 – 3.58 (m, 2H), 3.40 (s, 3H).

Synthesis of 19.

Compound **18** (533.4 mg, 1.0 mmol) in dichloromethane (30.0 mL) were mixed with 2-methylpyrrole (200.0μL, 2.3 mmol) and trifluoroacetic acid (30.0 μL) under Ar atmosphere. After stirring for overnight, *p*-chloranil (245.4 mg, 1.0 mmol) was added to the above solution. After stirred for another 2 h, the solvent was removed by rotary evaporator and the residue was purified by alumina column chromatography (hexane/dichloromethane = 2/1). The orange-brown band was collected and evaporated to give the product as yellow powder. Yield: 583.6 mg (84.5%). ^1H NMR (400 MHz, $CDCl_3$) δ = 8.00 – 7.95 (m, 2H), 7.72 – 7.69 (m, 2H), 7.40 (t, J = 8.5 Hz, 2H), 7.32 – 7.29 (m, 4H), 7.18 – 7.16 (m, 4H), 6.04 – 5.92 (m, 4H), 4.30 (t, J = 4.2 Hz, 2H), 3.98 (t, J = 4.8 Hz, 2H), 3.85 – 3.79 (m, 2H), 3.77 – 3.70 (m, 4H), 3.61 – 3.59 (m, 2H), 3.41 (s, 3H), 2.50 (s, 6H).

Synthesis of Z-5.

Compound **19** (600.1 mg, 1.0 mmol) in dichloromethane (20.0 mL) was mixed with zinc acetate (110.4 mg, 0.5 mmol) in methanol (10.0 mL). After stirring for overnight, the reaction solution was reduced by a rotary evaporator to afford the brown precipitate, which was recrystallized, filtered and rinsed with cold methanol.

Then the crude product was further purified by silica gel column chromatography (dichloromethane/ n-hexane = 2/1, v/v) to give **Z-5** as orange-brown solid. Yield: 1150.6 mg (91.1%). ^{1}H NMR (400 MHz, $CDCl_3$) δ = 8.04 – 8.02 (m, 4H), 7.77 – 7.72 (m, 4H), 7.45 (d, *J* = 4.2 Hz, 4H), 7.36 – 7.31 (m, 8H), 7.20 – 7.18 (m, 4H), 6.20 – 6.12 (m, 8H), 4.34 – 4.30 (m, 4H), 4.01 – 3.97 (m, 4H), 3.84 – 3.82 (m, 4H), 3.77 – 3.75 (m, 4H), 3.73 – 3.71 (m, 4H), 3.62 – 3.59 (m, 4H), 3.42 (s, 6H), 2.42 (s, 12H). ^{13}C NMR (100 MHz, $CDCl_3$) δ = 159.17, 158.34, 141.48, 140.48, 137.67, 133.06, 133.01, 132.46, 131.12, 129.78, 127.22, 126.92, 125.49, 124.94, 117.95, 117.72, 114.57, 72.00, 70.94, 70.76, 70.64, 69.89, 67.60, 59.09, 29.72, 16.87. ESI-HRMS: $C_{76}H_{74}N_4O_8Zn$ = 1236.8318 (calc.); *m/z* = 1236.4841 (found).

Structural characterization

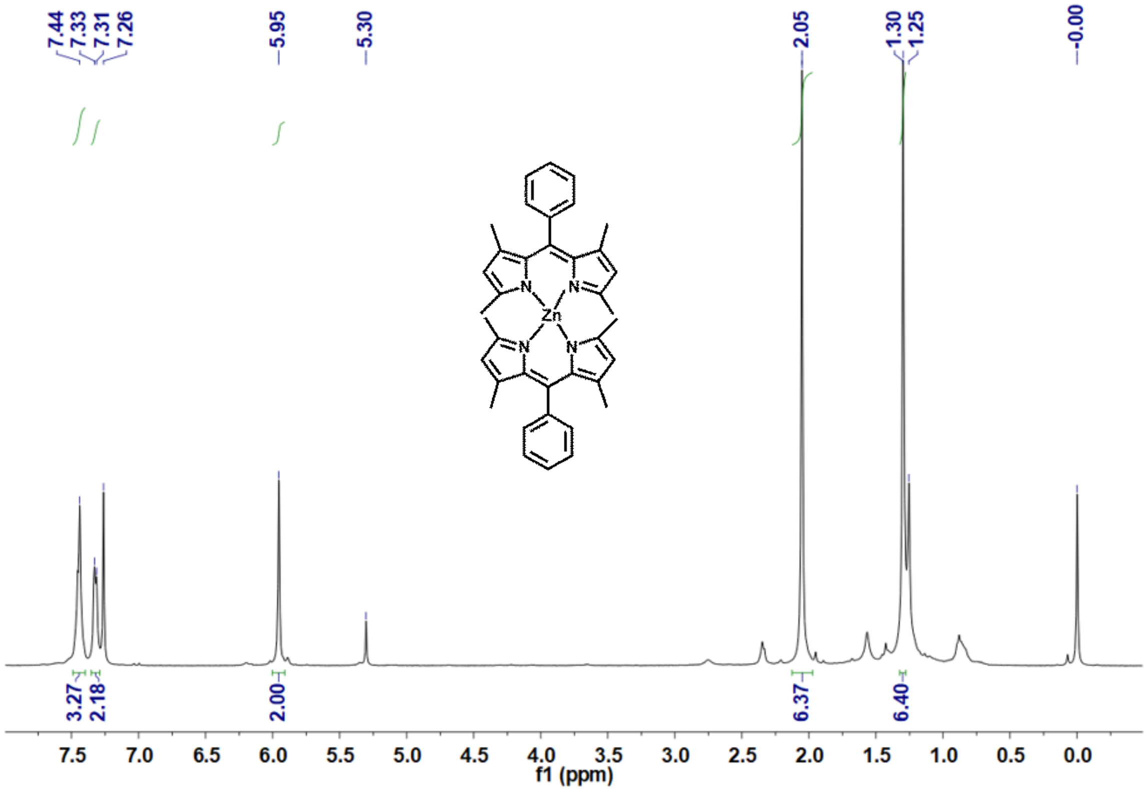

Supplementary Figure 2. ^{1}H NMR spectrum of **Z-1** (400 MHz, $CDCl_3$).

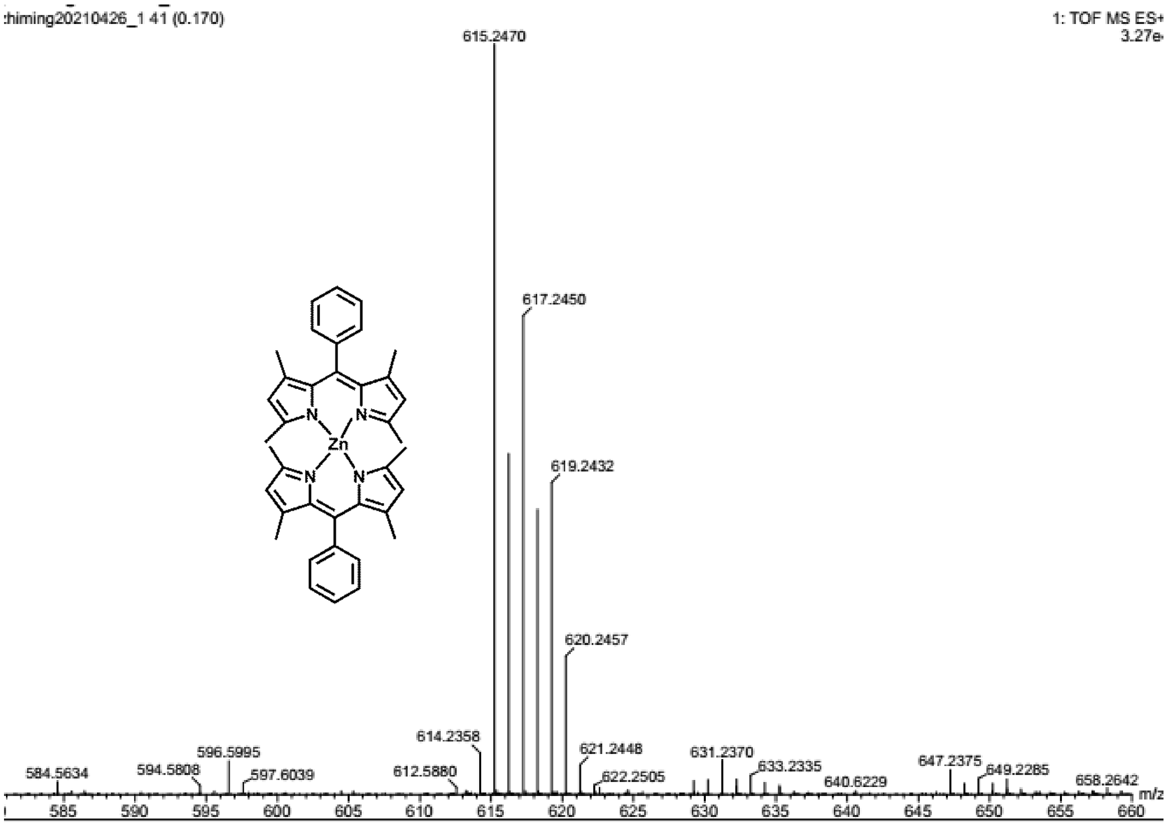

Supplementary Figure 3. HRMS ESI of **Z-1**

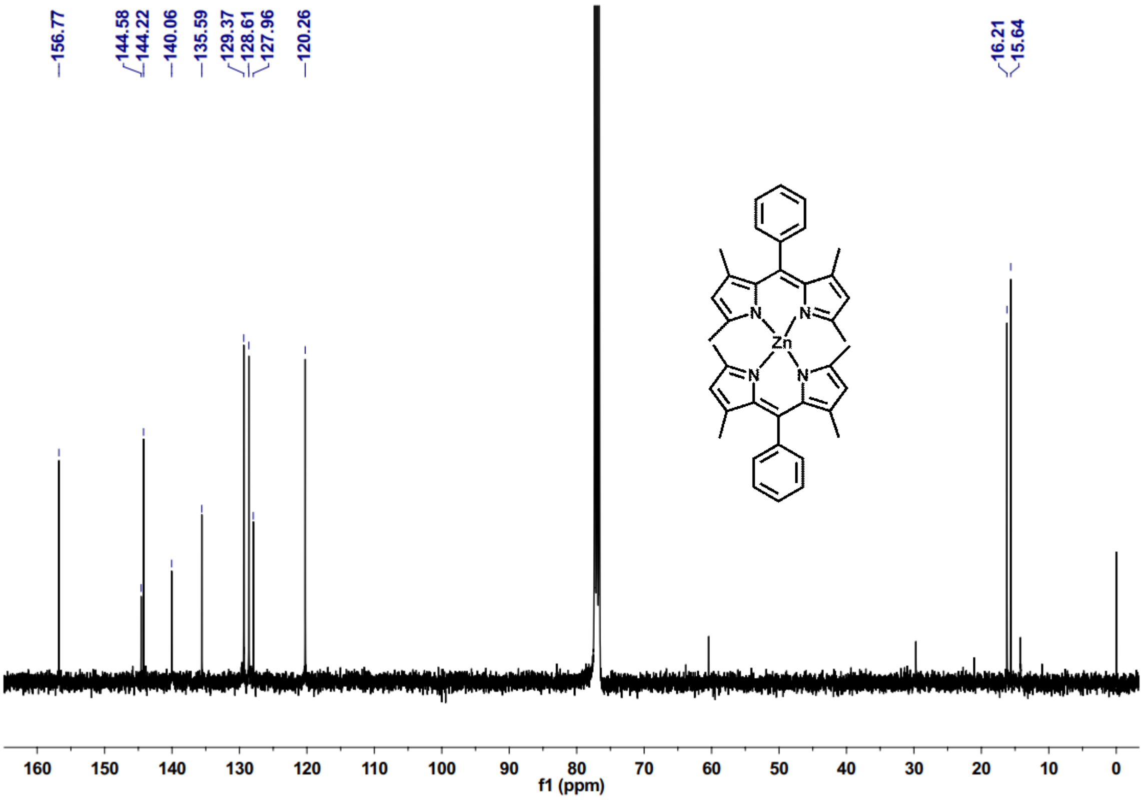

Supplementary Figure 4. ^{13}C NMR spectrum of **Z-1** (100 MHz, $CDCl_3$).

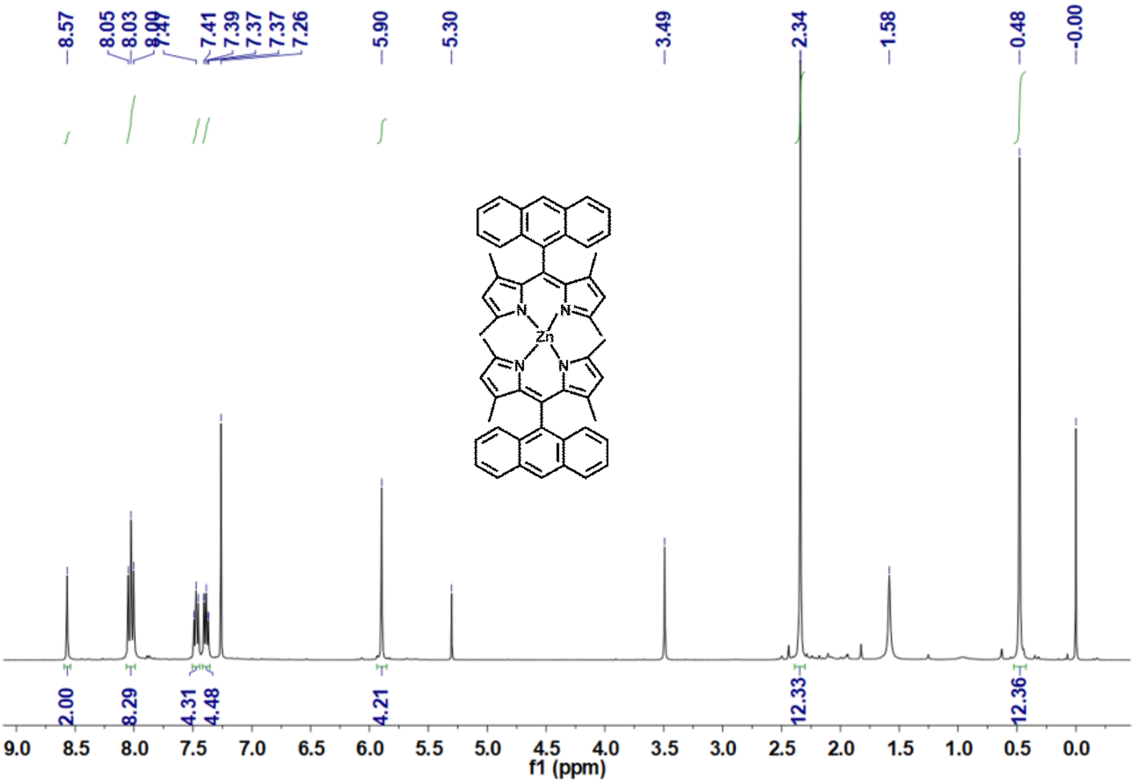

Supplementary Figure 5. ^{1}H NMR spectrum of **Z-2** (400 MHz, $CDCl_3$).

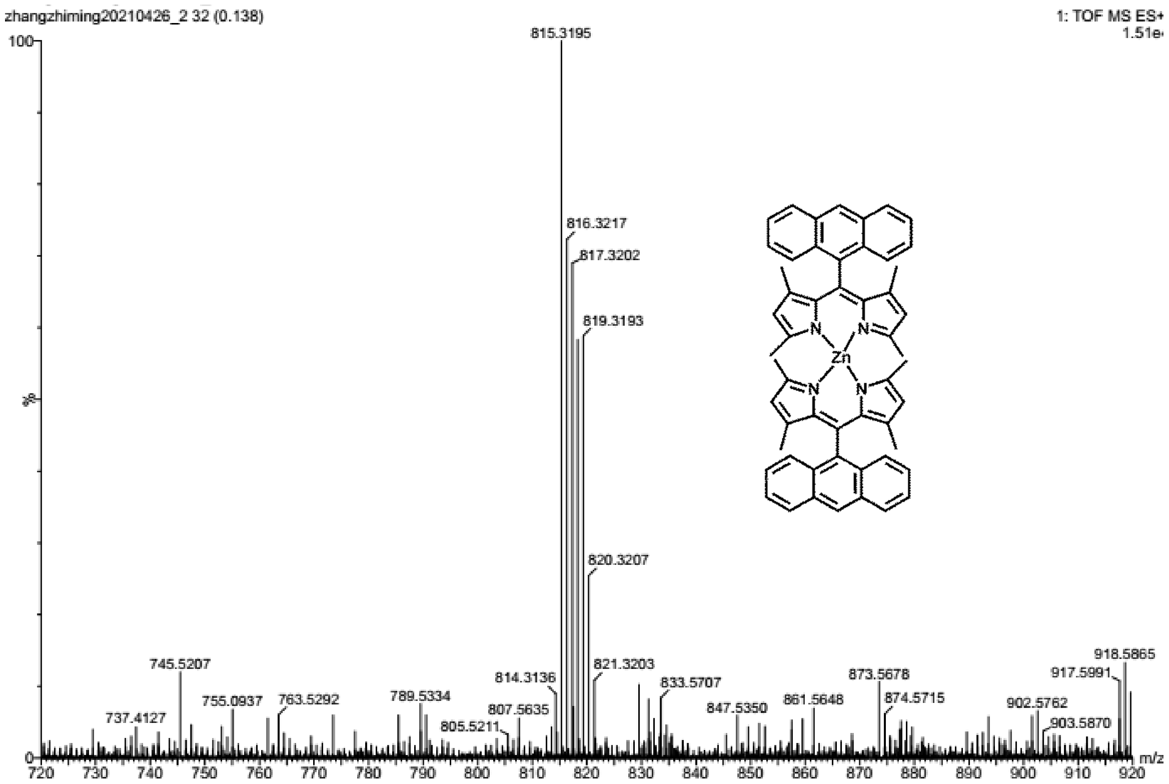

Supplementary Figure 6. HRMS ESI of **Z-2**.

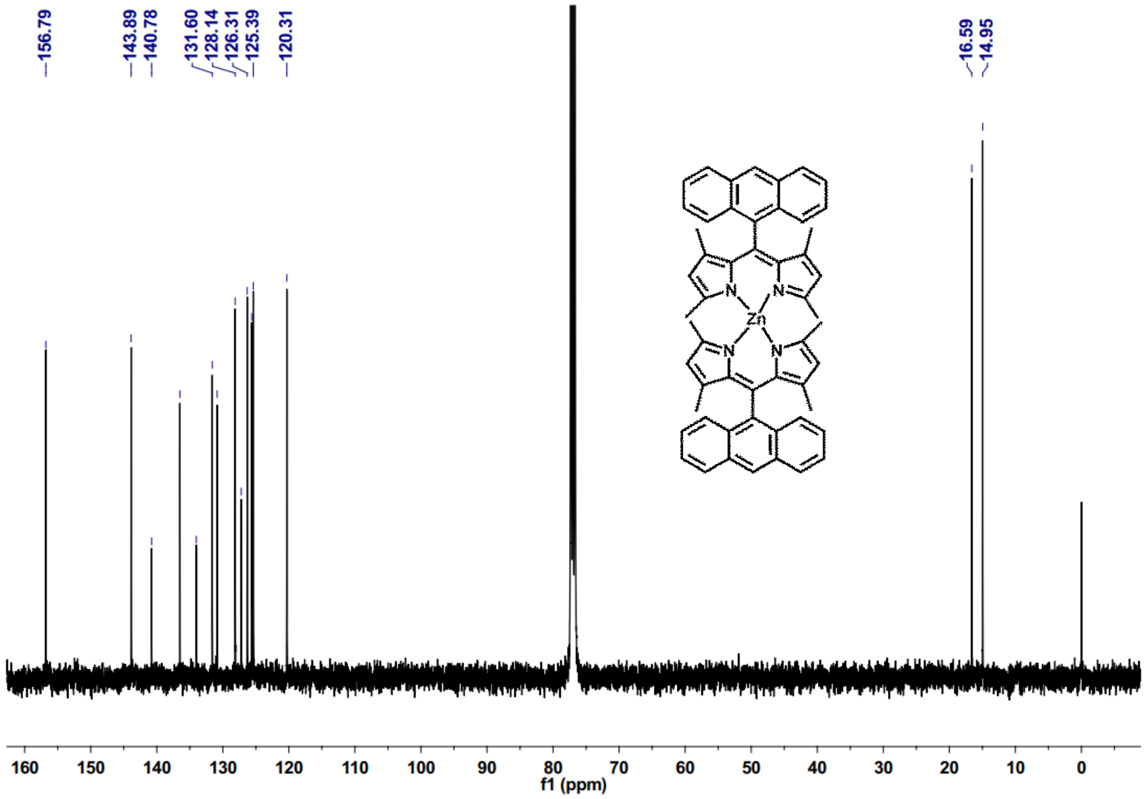

Supplementary Figure 7. ^{13}C NMR spectrum of **Z-2** (100 MHz, $CDCl_3$).

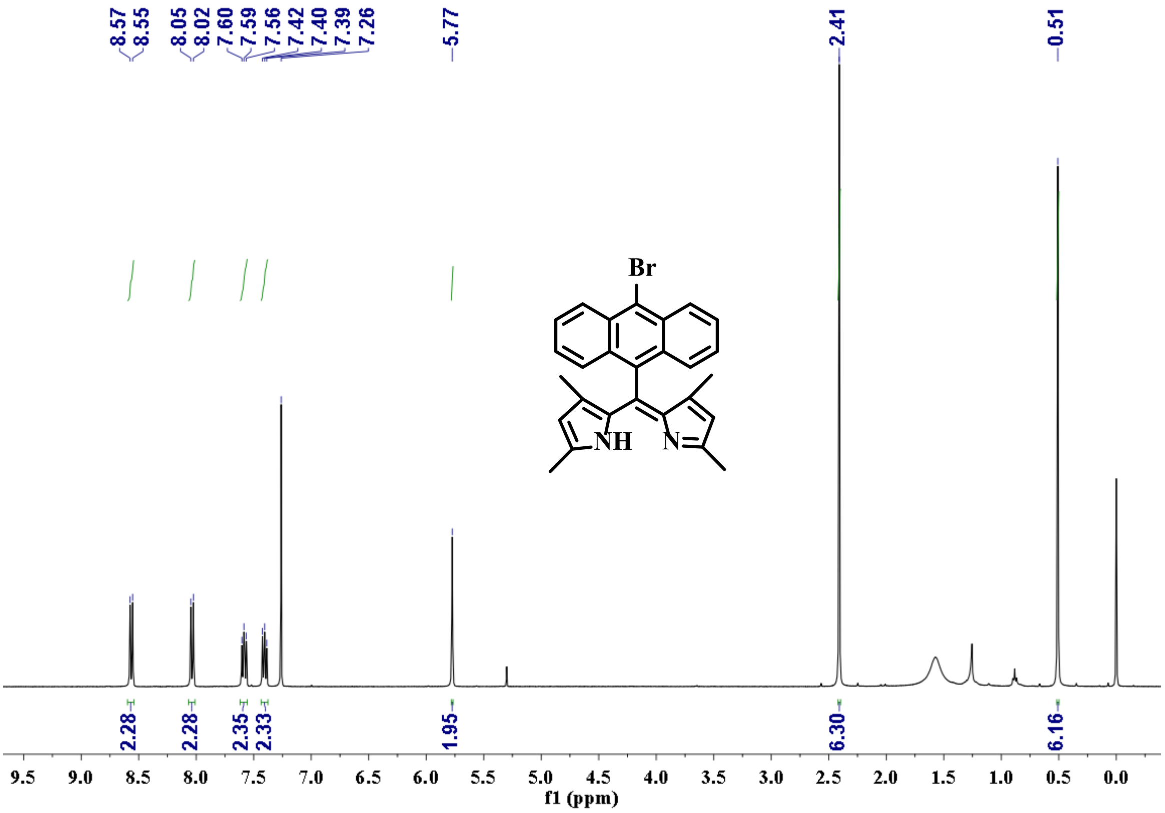

Supplementary Figure 8. ^1H NMR spectrum of **6** (400 MHz, $CDCl_3$).

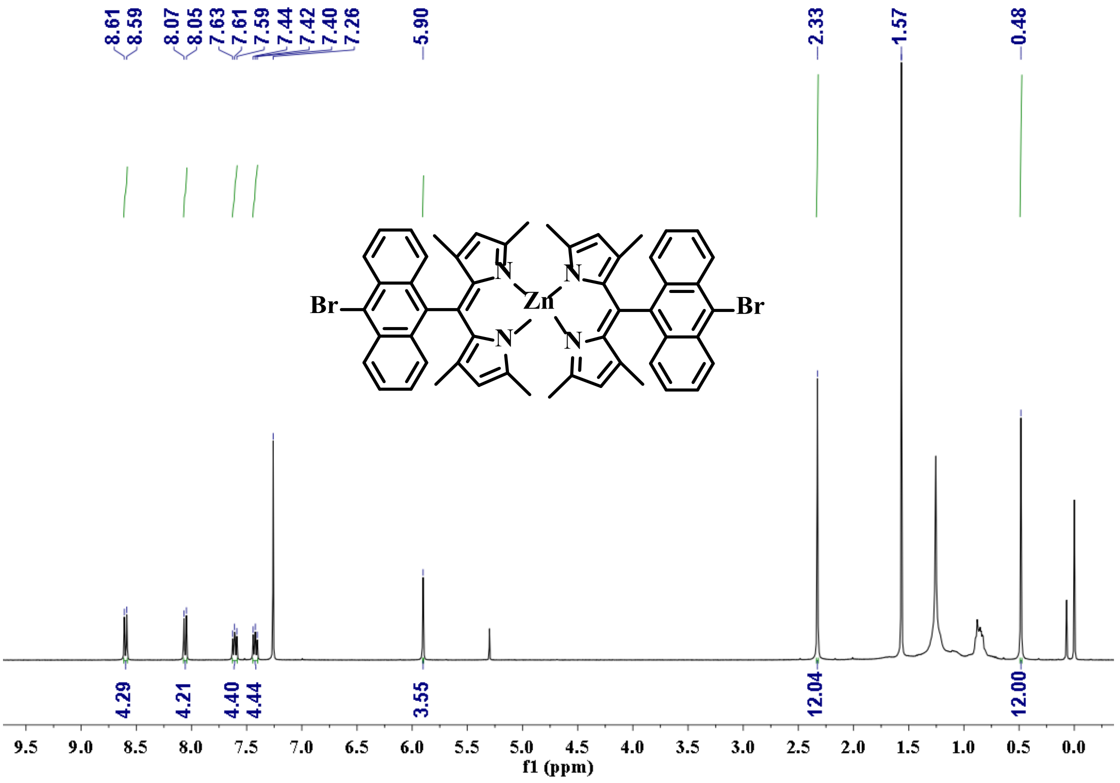

Supplementary Figure 9. ^1H NMR spectrum of **7** (400 MHz, $CDCl_3$).

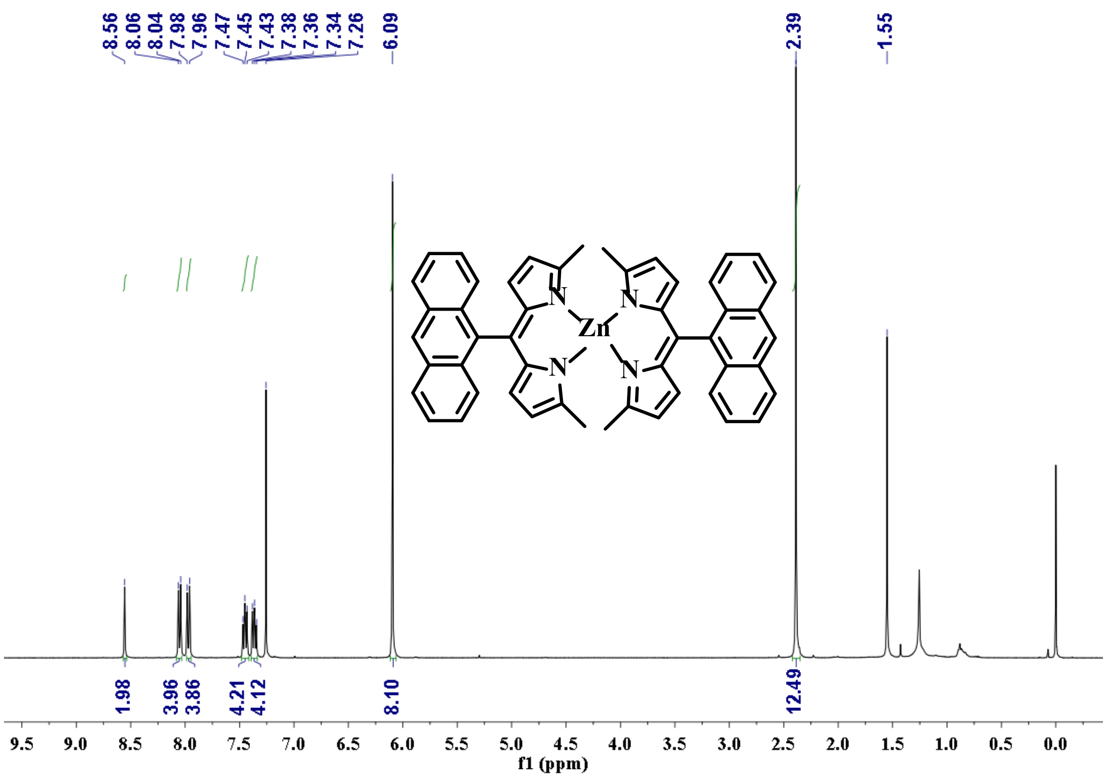

Supplementary Figure 10. ^{1}H NMR spectrum of **Z-3** (400 MHz, $CDCl_3$).

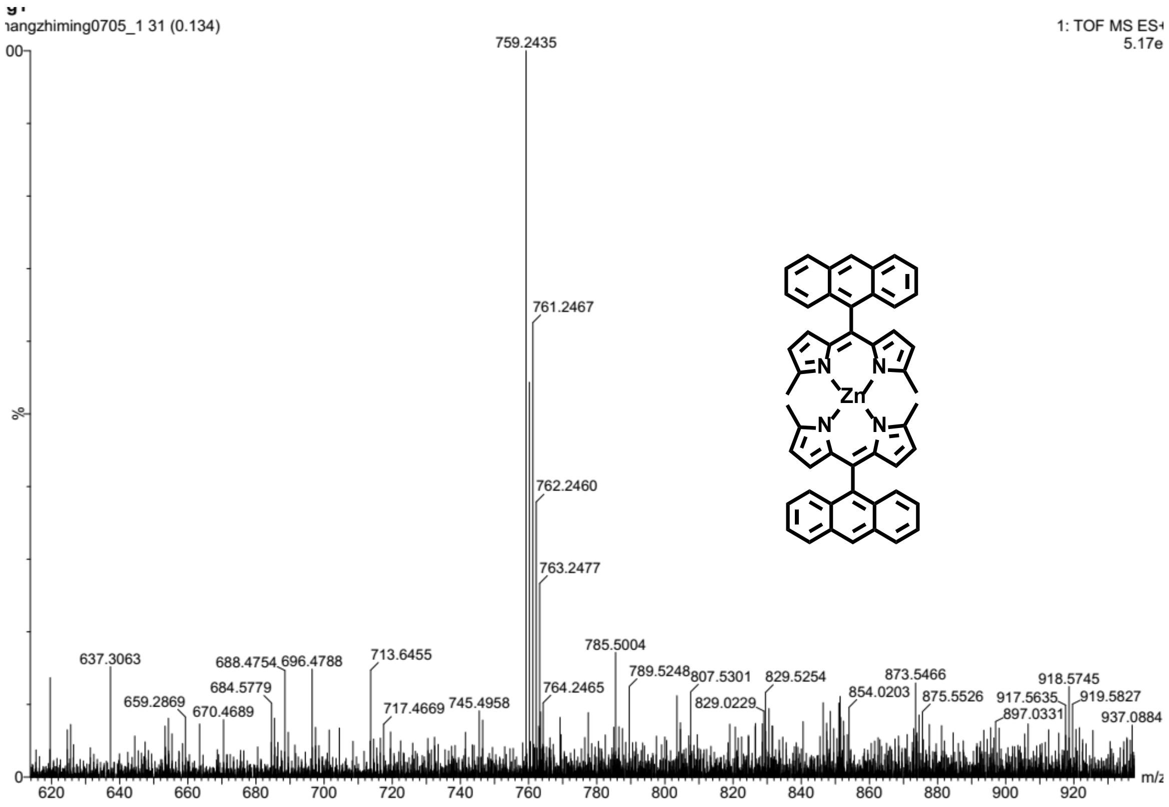

Supplementary Figure 11. HRMS ESI of **Z-3**.

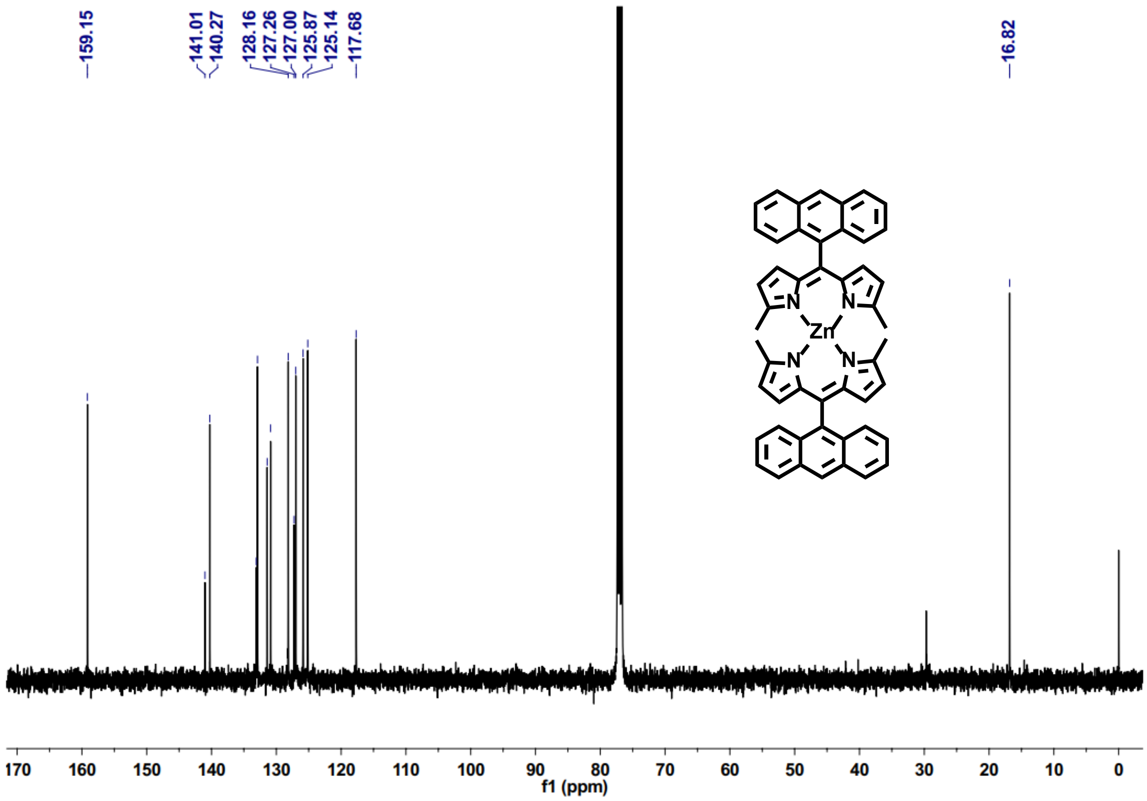

Supplementary Figure 12. ^{13}C NMR spectrum of **Z-3** (100 MHz, $CDCl_3$).

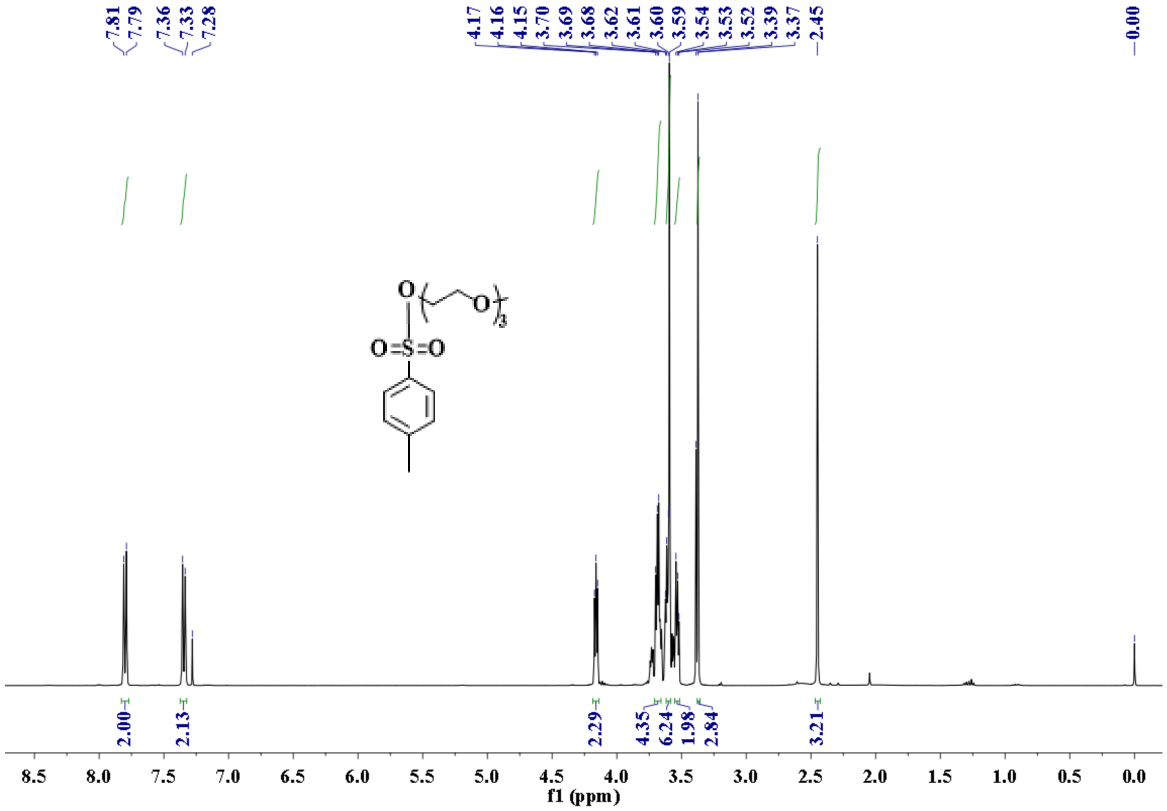

Supplementary Figure 13. ^{1}H NMR spectrum of **10** (400 MHz, $CDCl_3$).

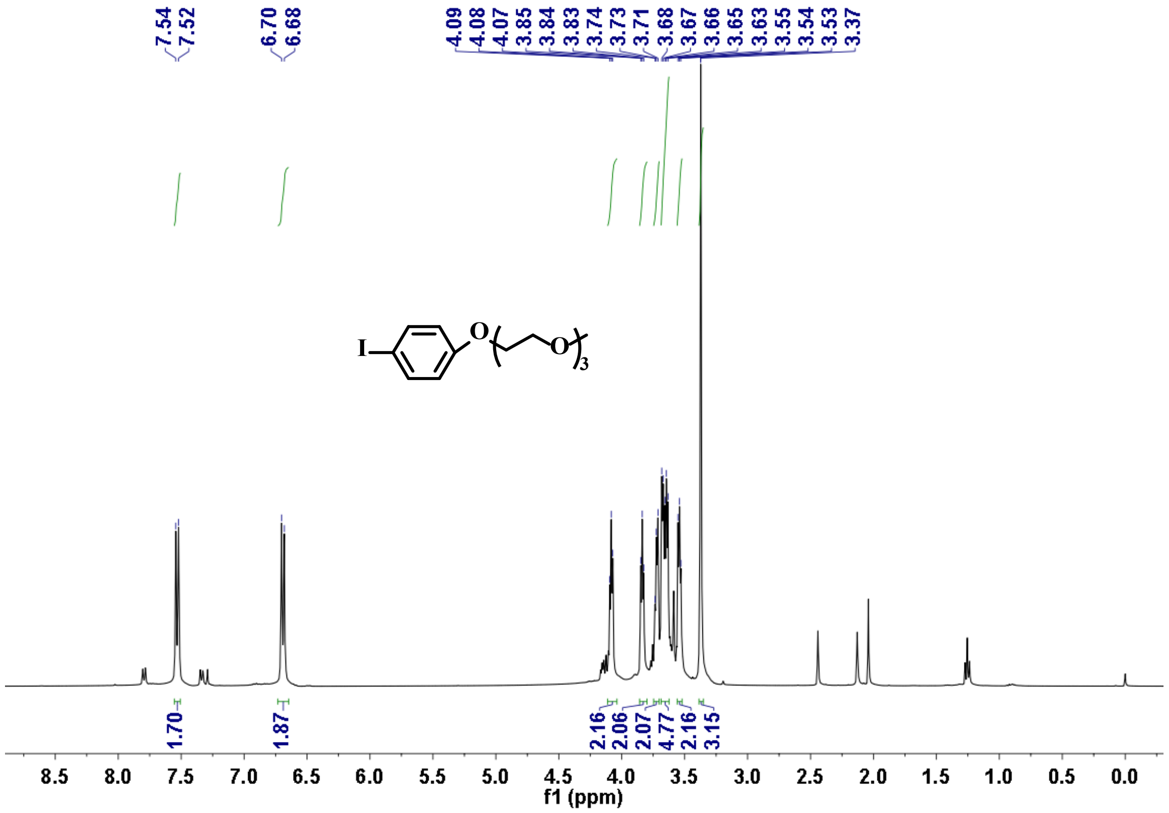

Supplementary Figure 14. ^{1}H NMR spectrum of **11** (400 MHz, $CDCl_3$).

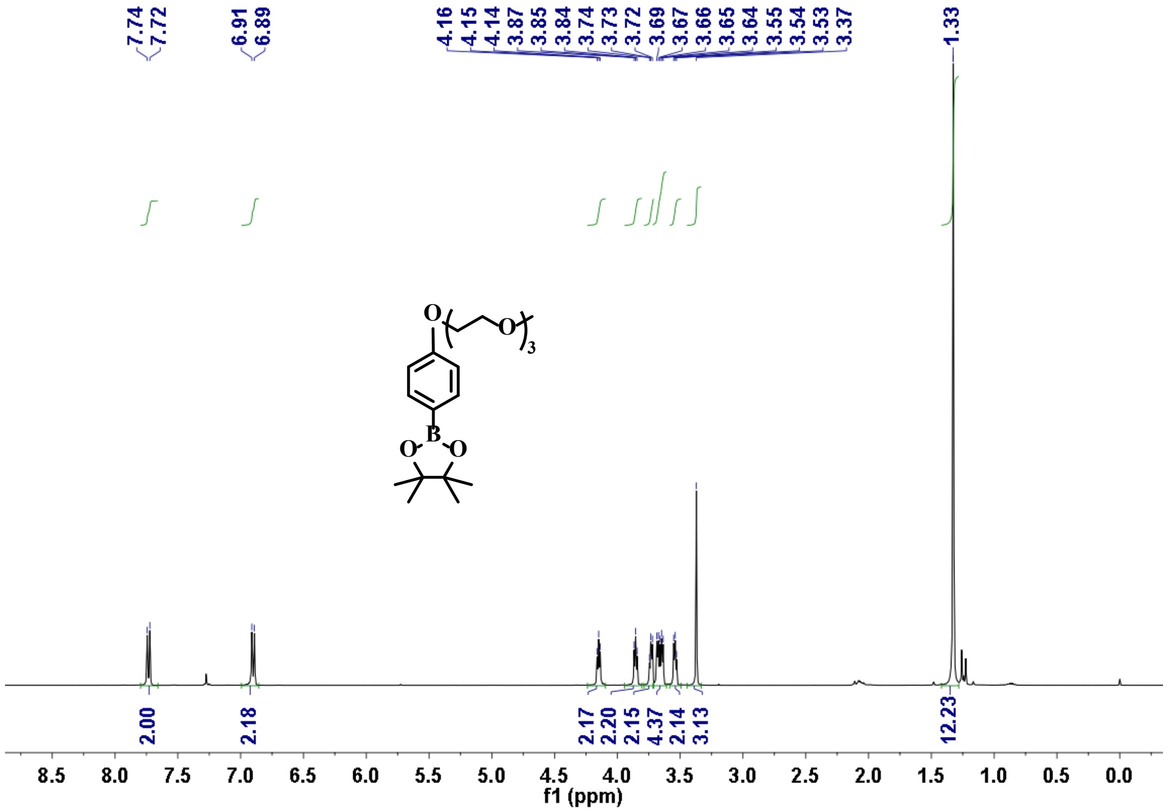

Supplementary Figure 15. ^{1}H NMR spectrum of **12** (400 MHz, $CDCl_3$).

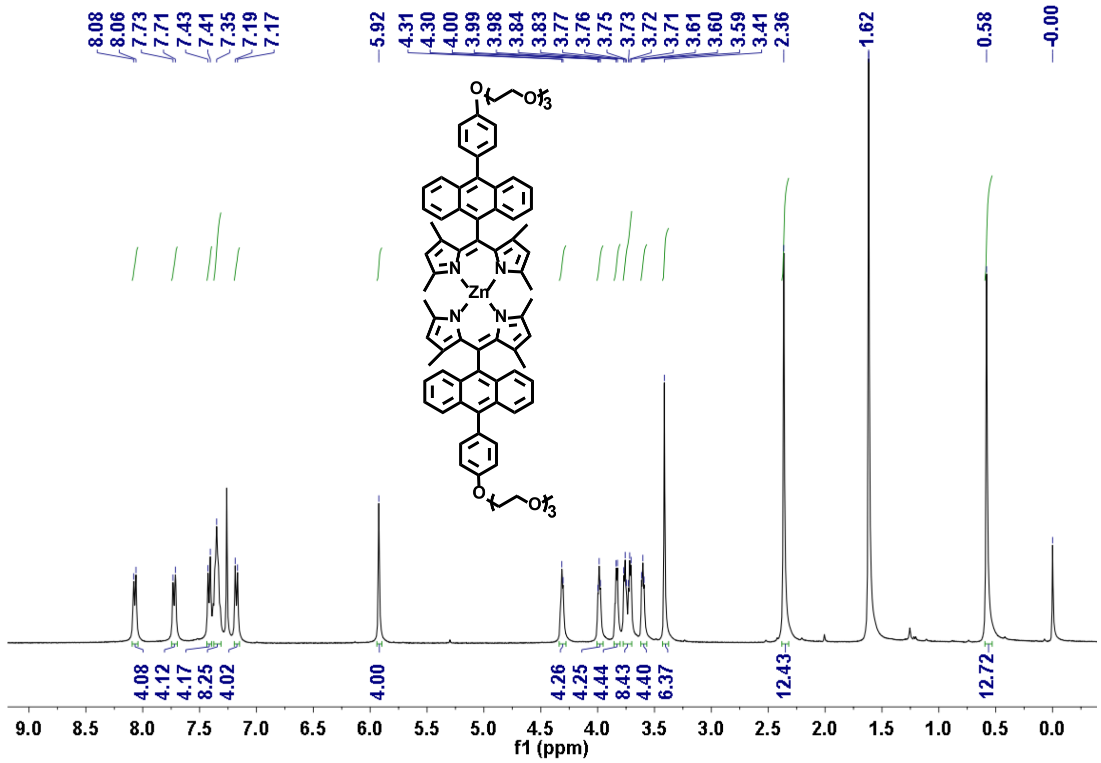

Supplementary Figure 16. ^{1}H NMR spectrum of **Z-4** (400 MHz, $CDCl_3$).

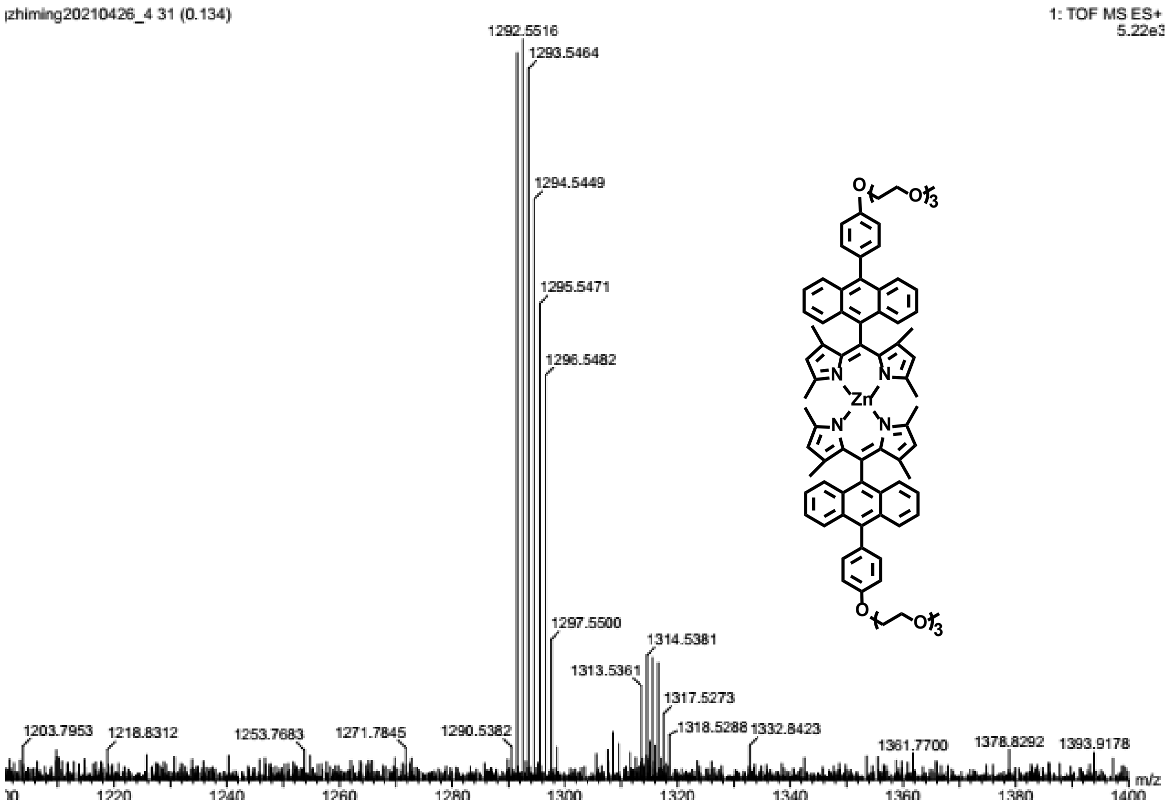

Supplementary Figure 17. HRMS ESI of **Z-4**.

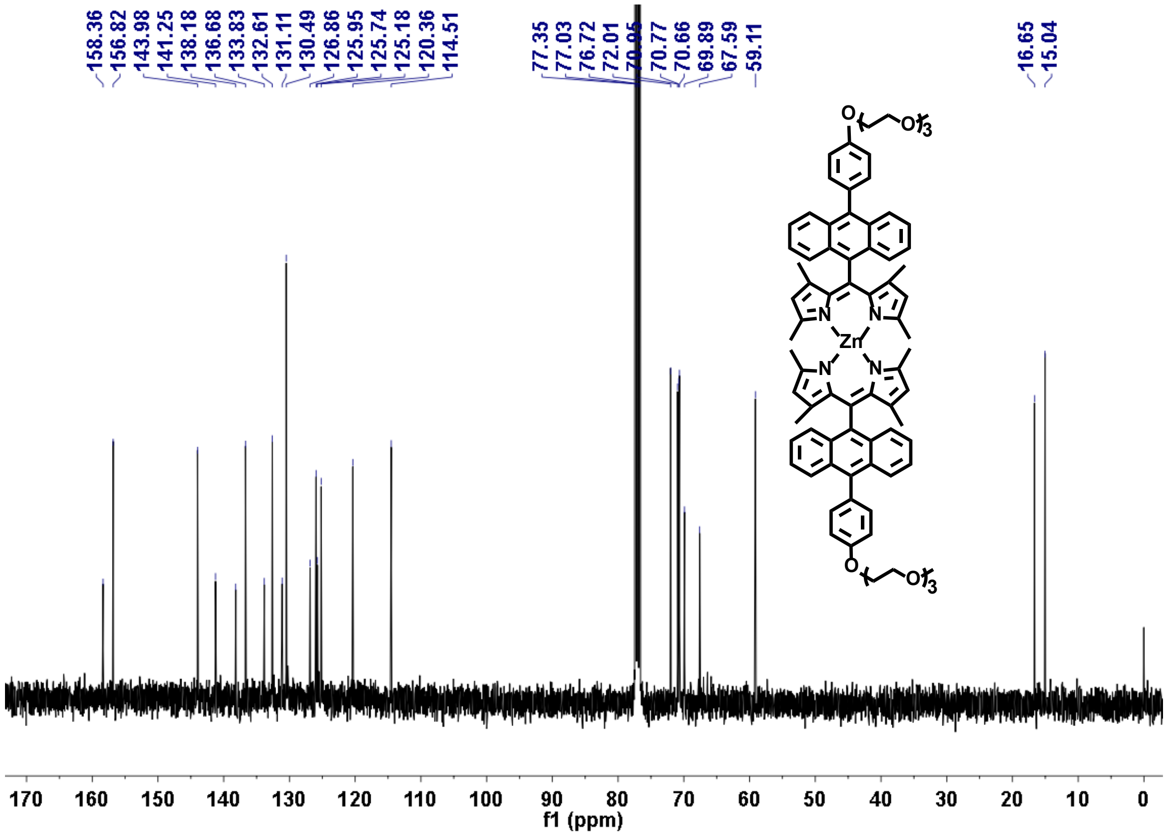

Supplementary Figure 18. ^{13}C NMR spectrum of **Z-4** (400 MHz, $CDCl_3$).

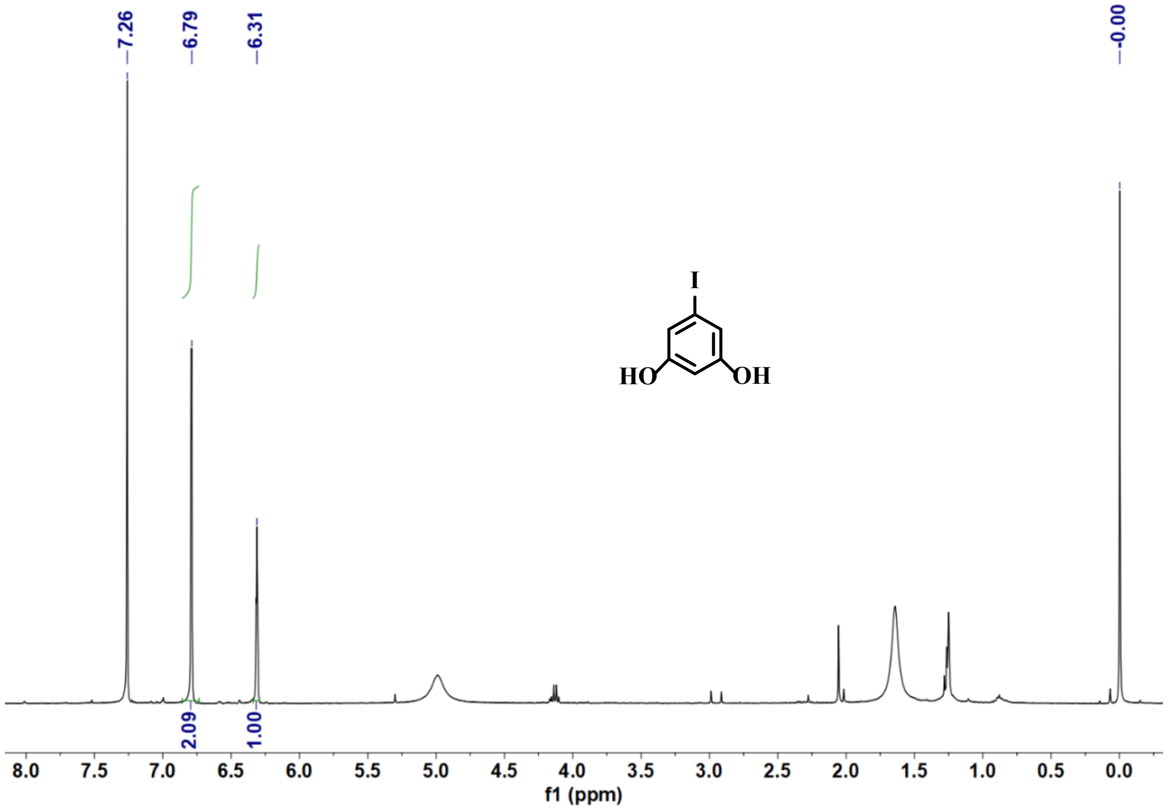

Supplementary Figure 19. ^{1}H NMR spectrum of **14** (400 MHz, $CDCl_3$).

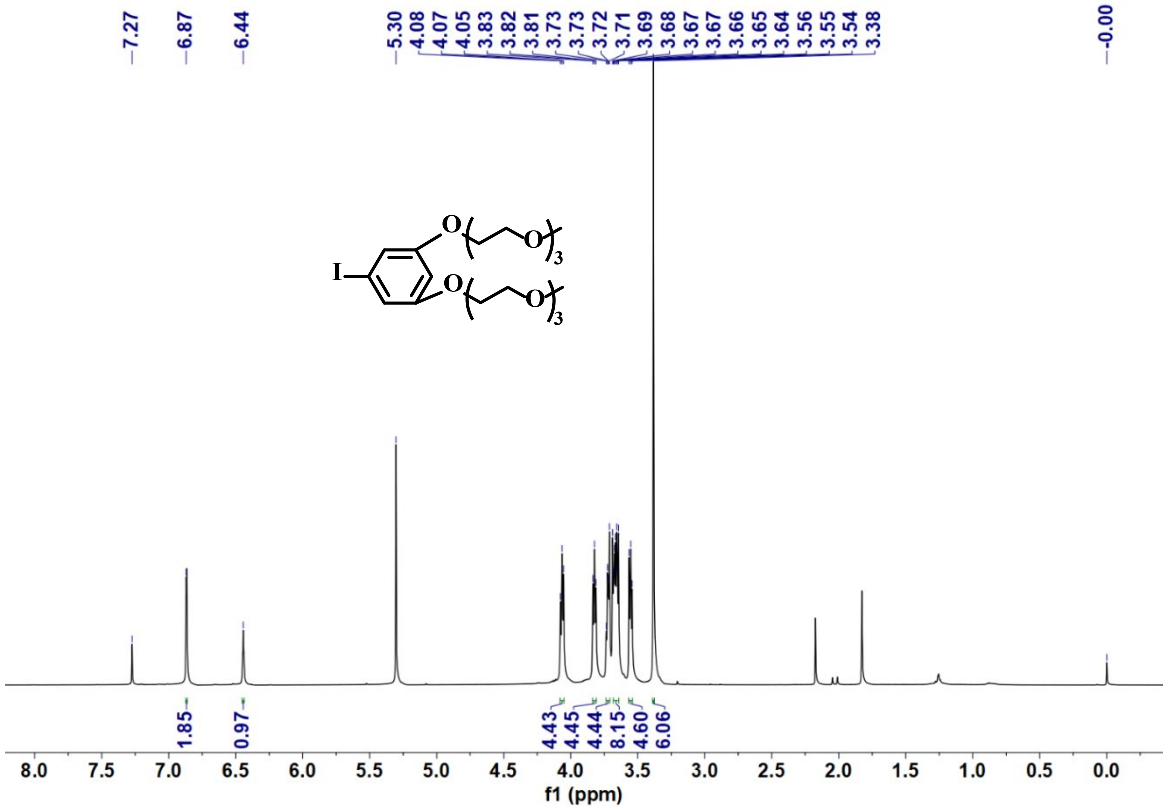

Supplementary Figure 20. ^{1}H NMR spectrum of **15** (400 MHz, $CDCl_3$).

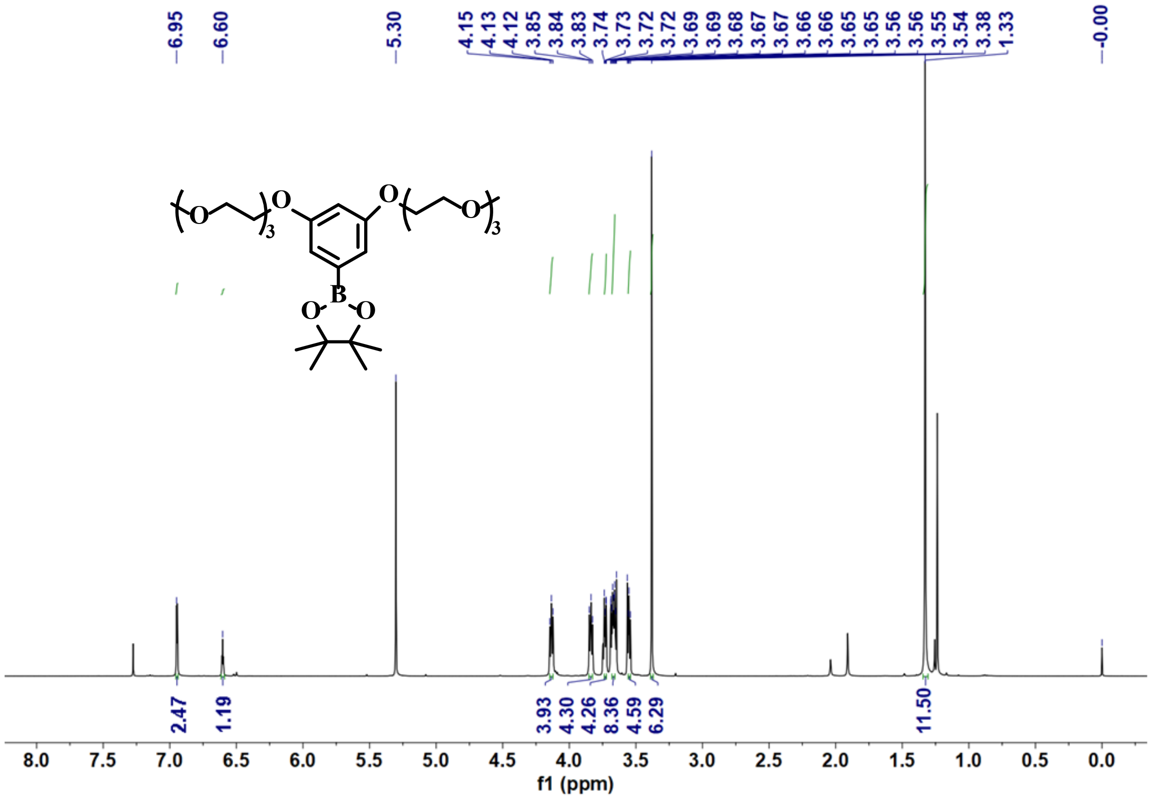

Supplementary Figure 21. ^{1}H NMR spectrum of **16** (400 MHz, $CDCl_3$).

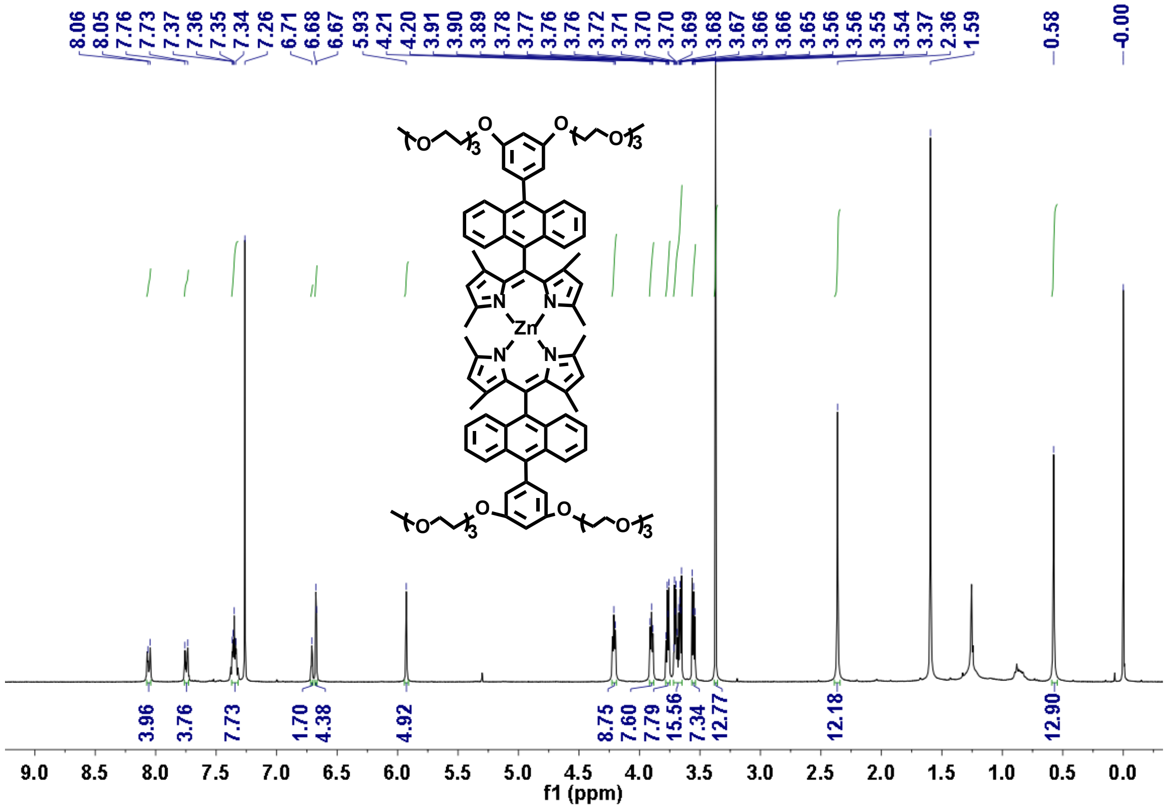

Supplementary Figure 22. ^{1}H NMR spectrum of **Z-6** (400 MHz, $CDCl_3$).

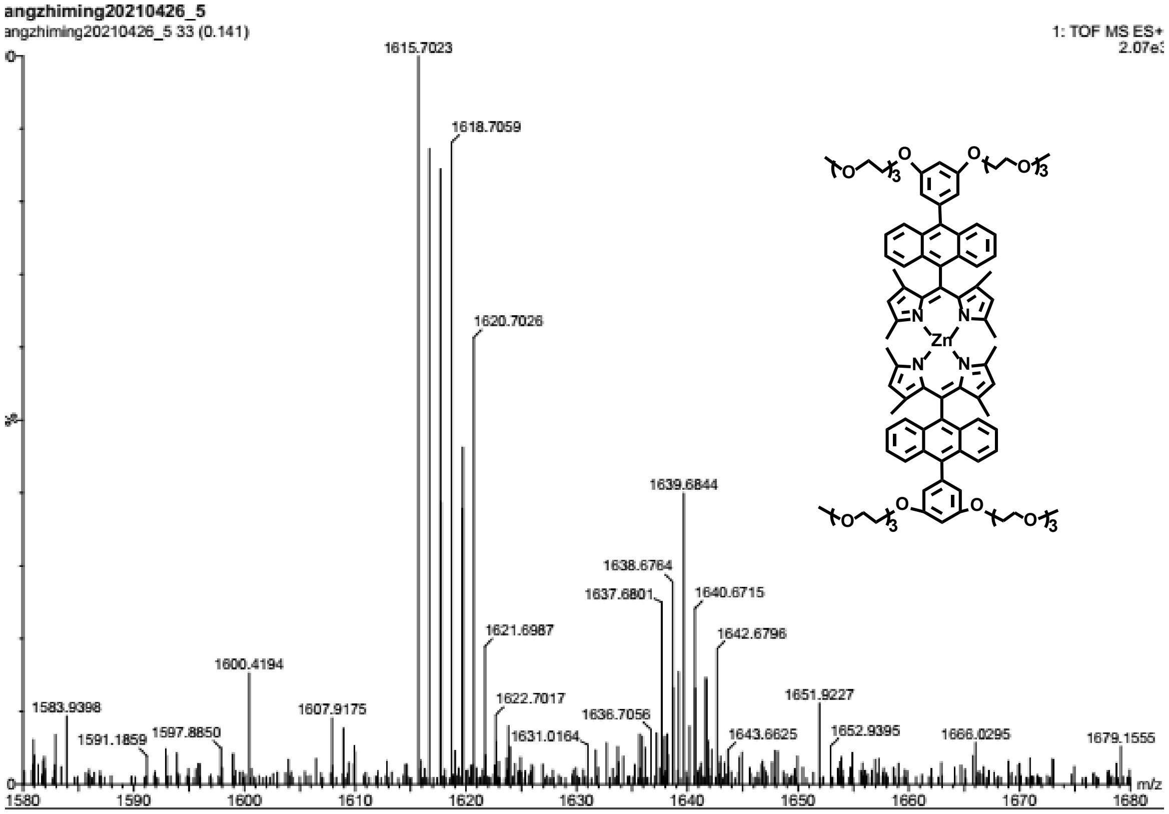

Supplementary Figure 23. HRMS ESI of **Z-6**.

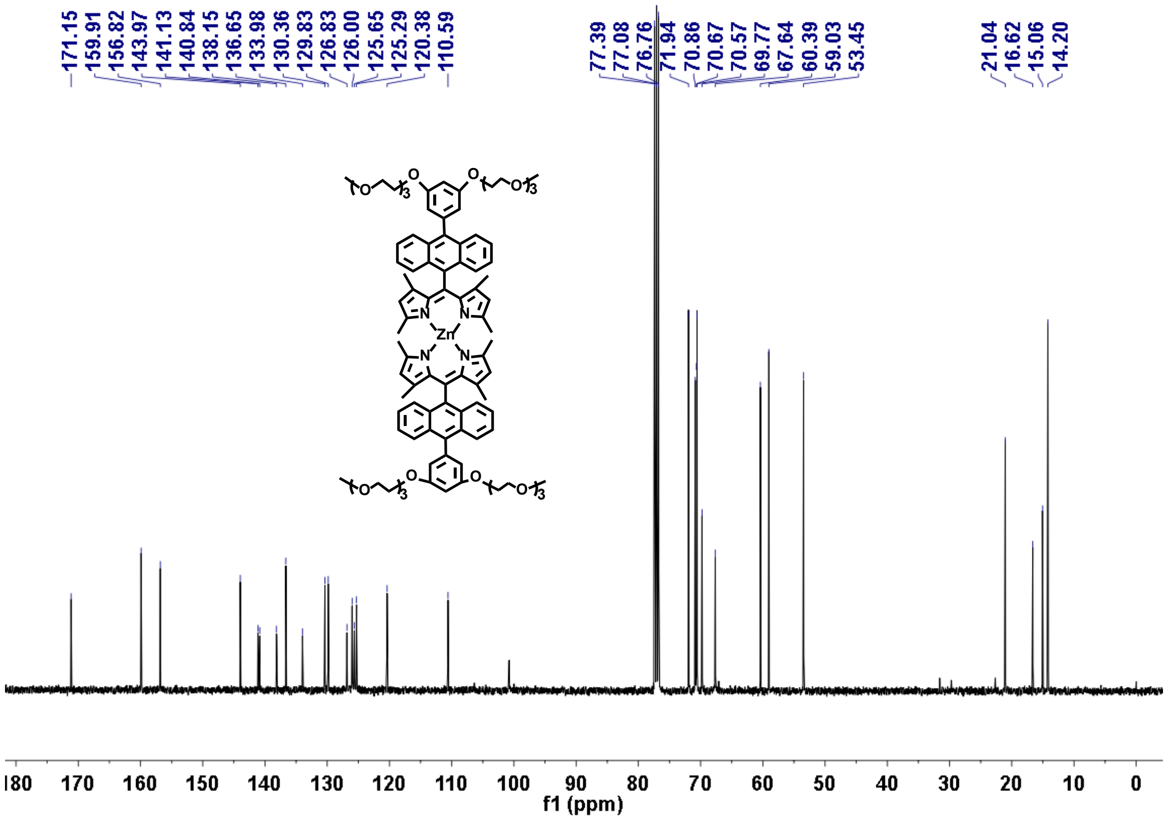

Supplementary Figure 24. ^{13}C NMR spectrum of **Z-6** (400 MHz, $CDCl_3$).

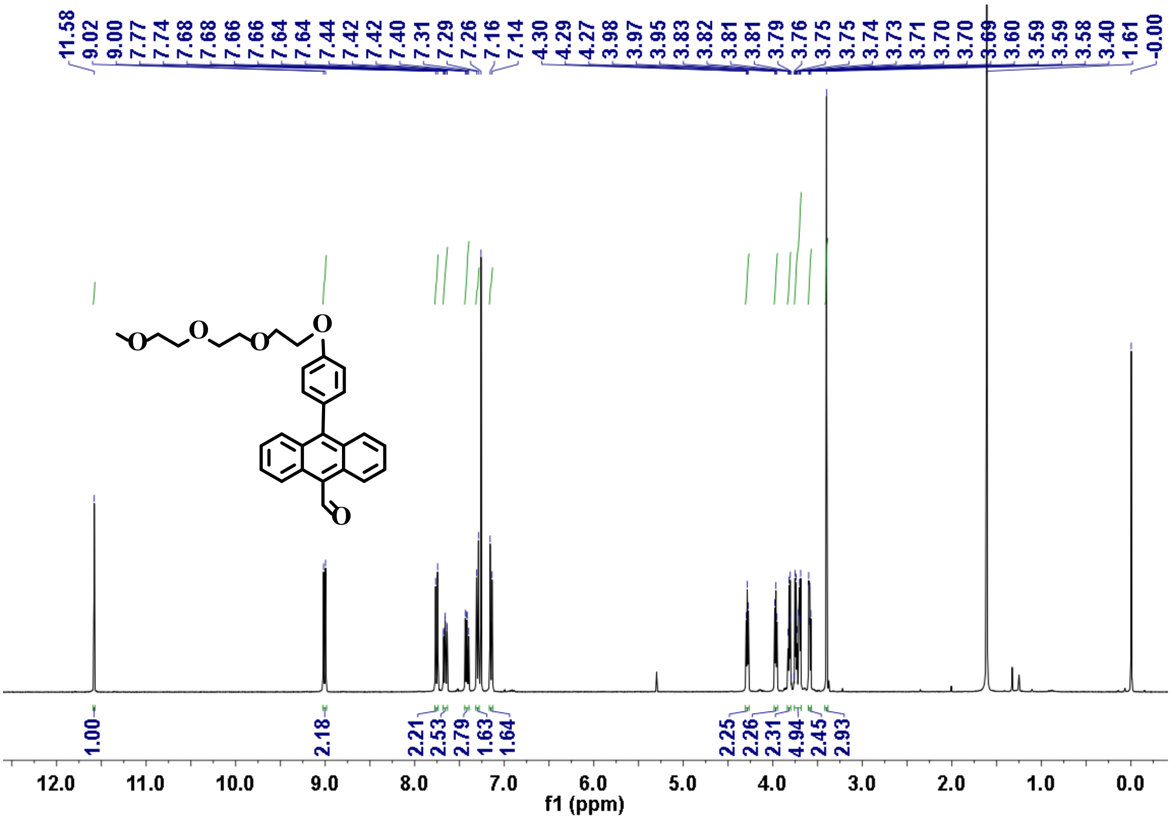

Supplementary Figure 25. ^{1}H NMR spectrum of **18** (400 MHz, $CDCl_3$).

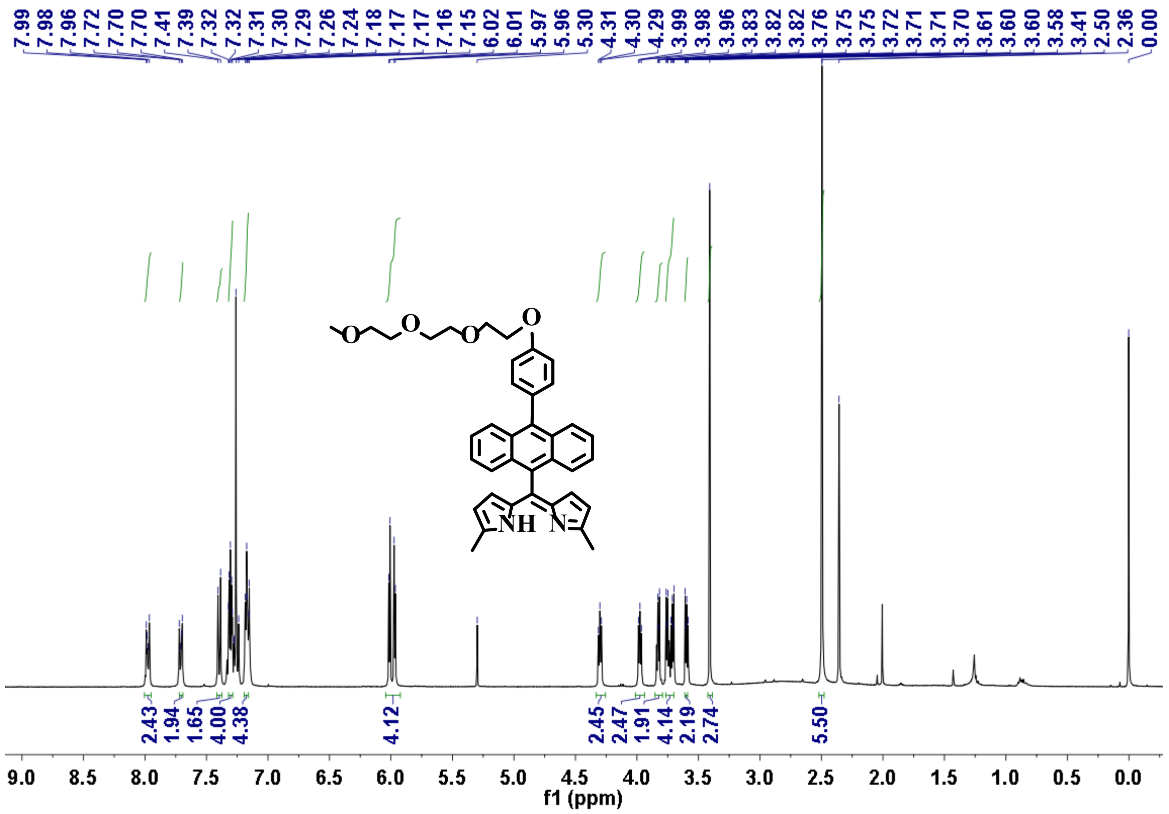

Supplementary Figure 26. ^{1}H NMR spectrum of **19** (400 MHz, $CDCl_3$).

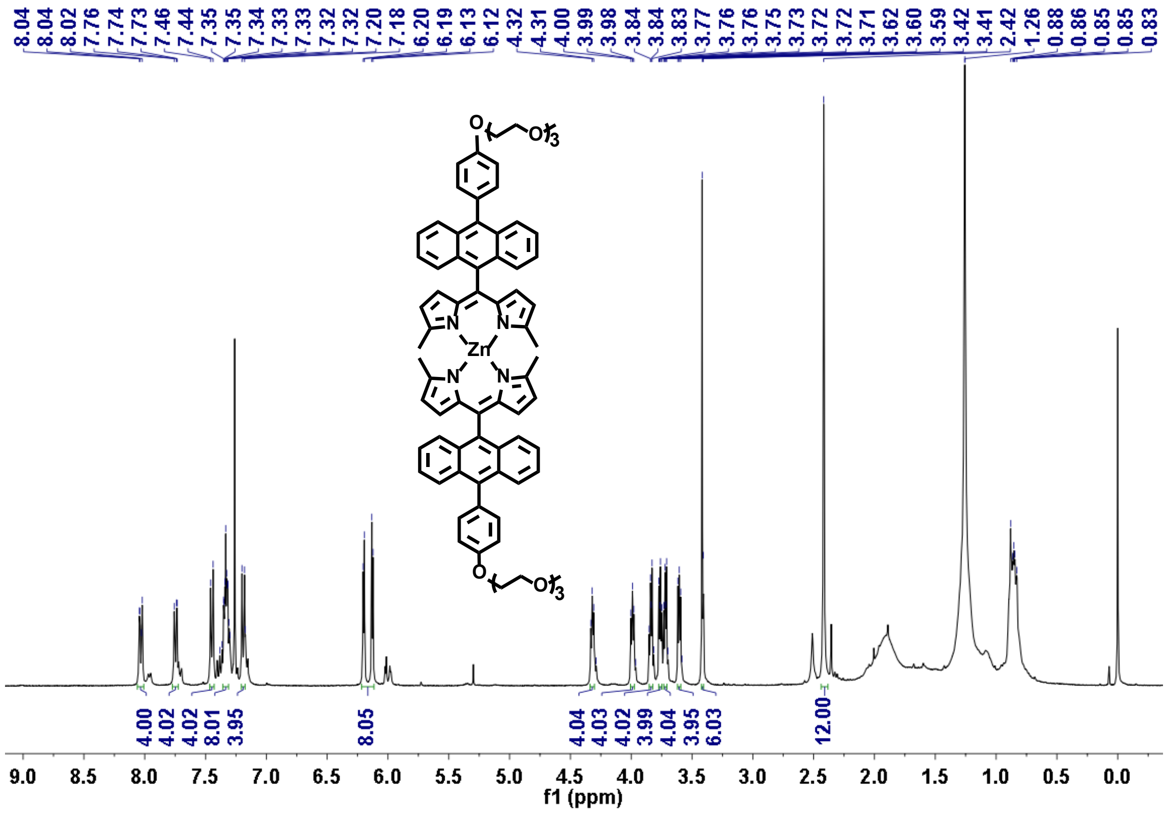

Supplementary Figure 27. ^{1}H NMR spectrum of **Z-5** (400 MHz, $CDCl_3$).

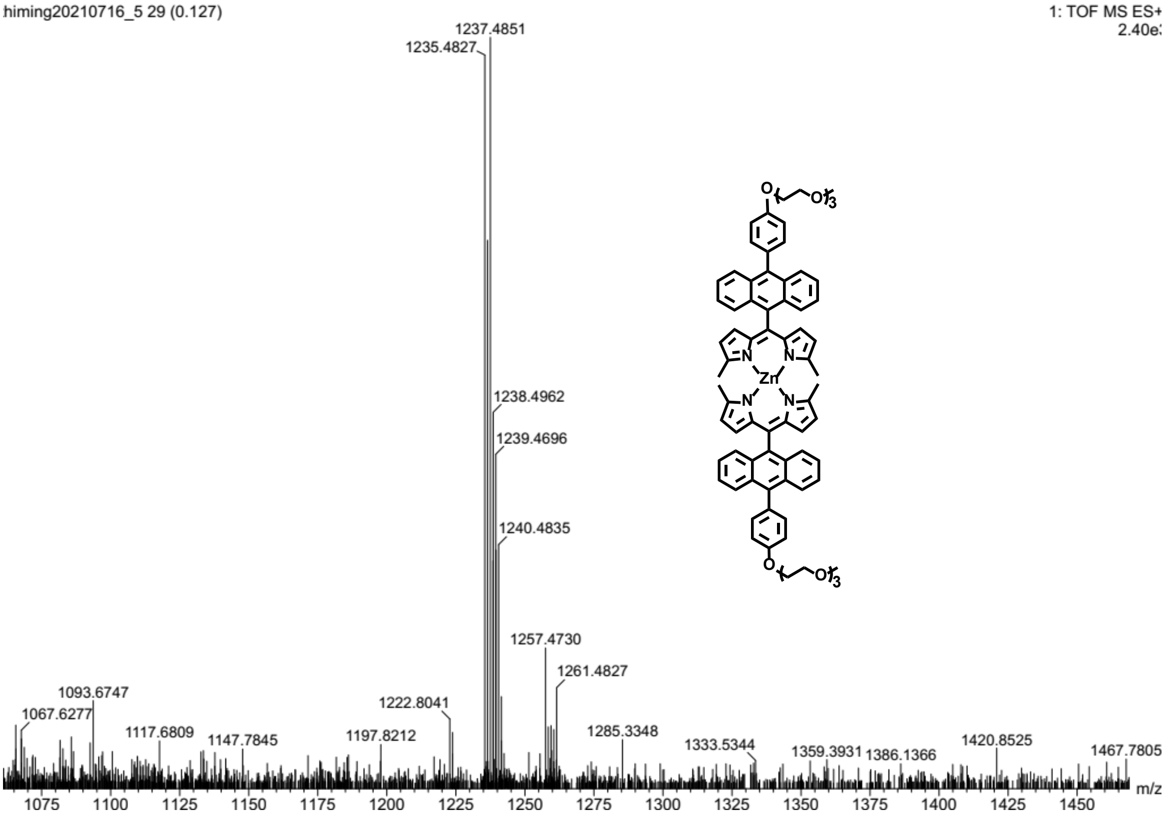

Supplementary Figure 28. HRMS ESI of **Z-5**.

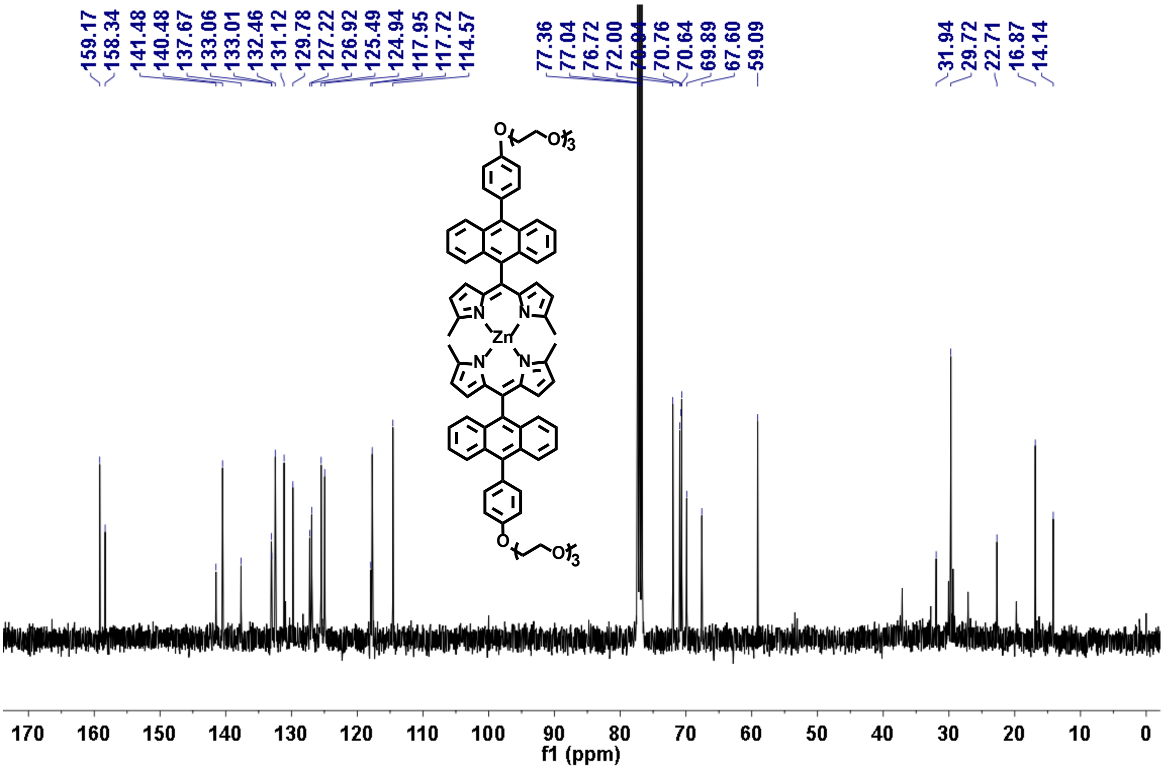

Supplementary Figure 29. ^{13}C NMR spectrum of **Z-5** (400 MHz, $CDCl_3$).

Supplementary Figures

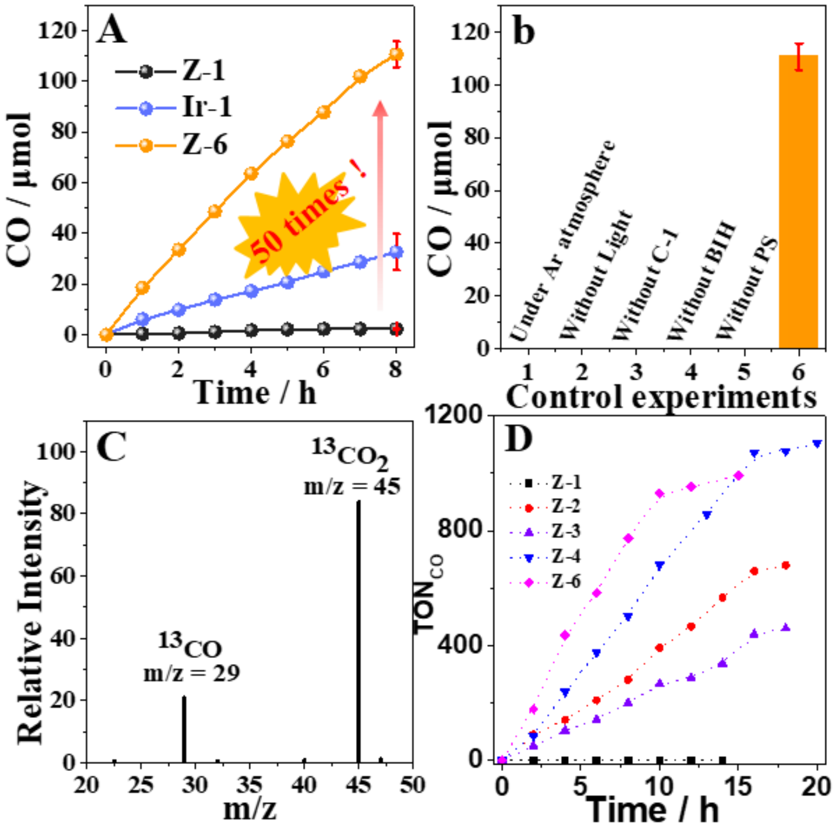

Supplementary Figure 30. Photocatalytic CO_2 reduction. (A) CO generation as a function of irradiation time with **Z-1**, **Z-6** and **Ir-1** as the PSs. (B) Control experiments. (C) GC-MS analysis of ^{13}CO generated under $^{13}CO_2$ atmosphere. Catalytic conditions: 0.5 µM PS, 10 µM C-1 ($[Fe(qpy)(OH_2)_2]^{2+}$), 20 mM BIH in 20 mL mixed solvent of CH_3CN/H_2O. Under CO_2 atmosphere, Xeon lamp with 420 nm filter as light source. (D) The time-conversion curves for determining the quantum yields of CO formation during the irradiation at 488 nm of CO_2-saturated CH_3CN (4.8 mL) containing 15 mM BIH, 5 µM PS, 200 µL H_2O and 10 µM C-1.

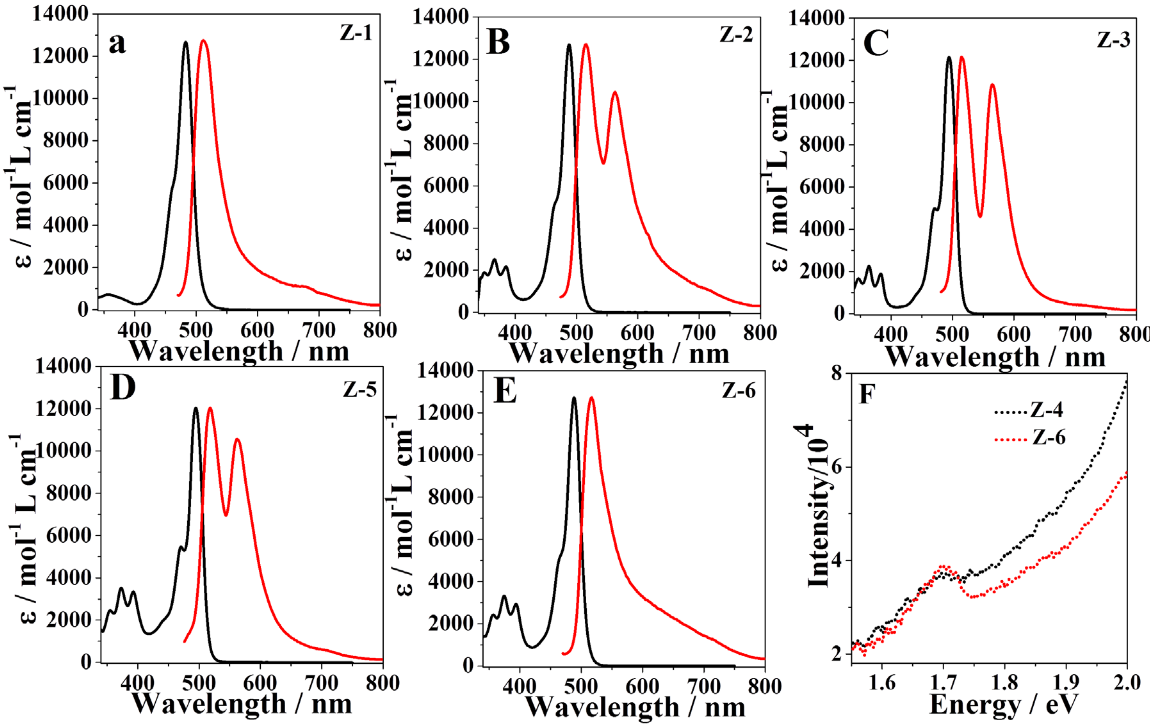

Supplementary Figure 31. UV-vis absorption and FL spectra of (A) **Z-1**, (B) **Z-2**, (C) **Z-3**, (D) **Z-5**, (E) **Z-6** at 298 K and (F) FL spectra of **Z-4** / **Z-6** in acetonitrile at 77 K, λ_{ex} = 455 nm, c_{ps} = 10.0 μM.

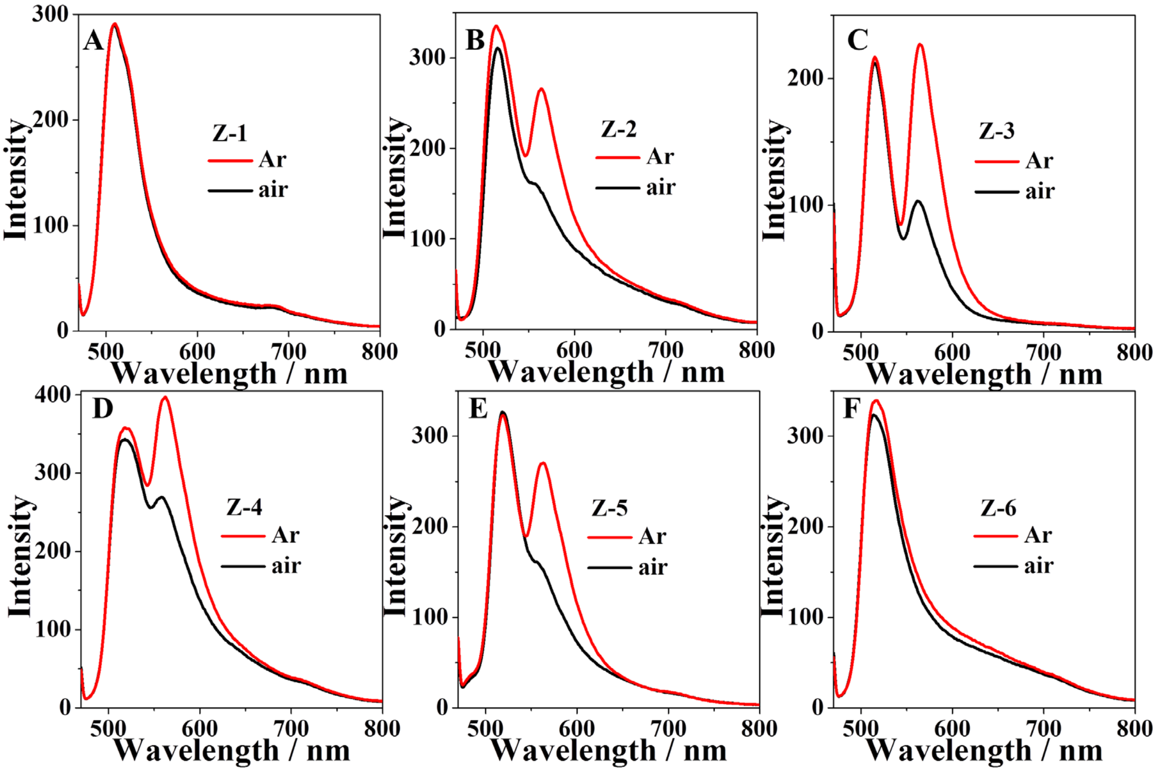

Supplementary Figure 32. FL spectra of PSs in acetonitrile under Ar (red) and air (black), λ_{ex} = 455 nm, c_{ps} = 10.0 μM.

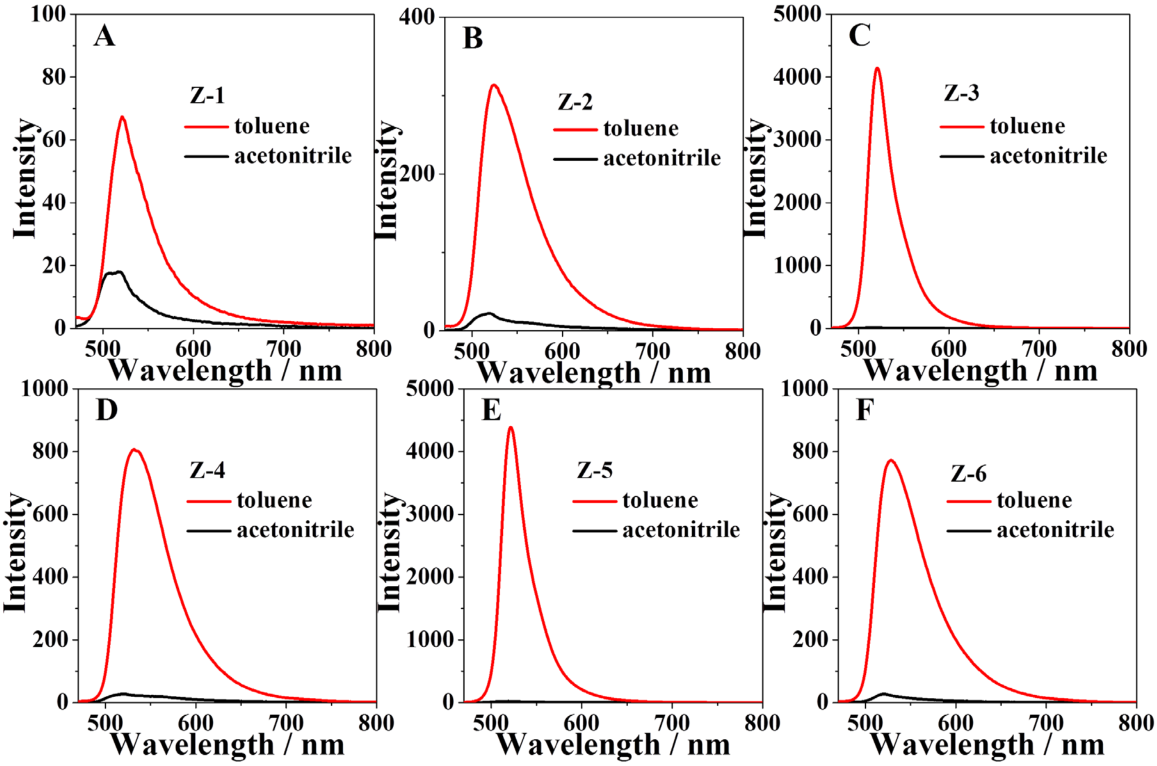

Supplementary Figure 33. FL spectra of PSs in toluene (red) and acetonitrile (black), λ_{ex} = 455 nm, c_{ps} = 10.0 μM. FL spectra of Zn PSs were performed under Ar.

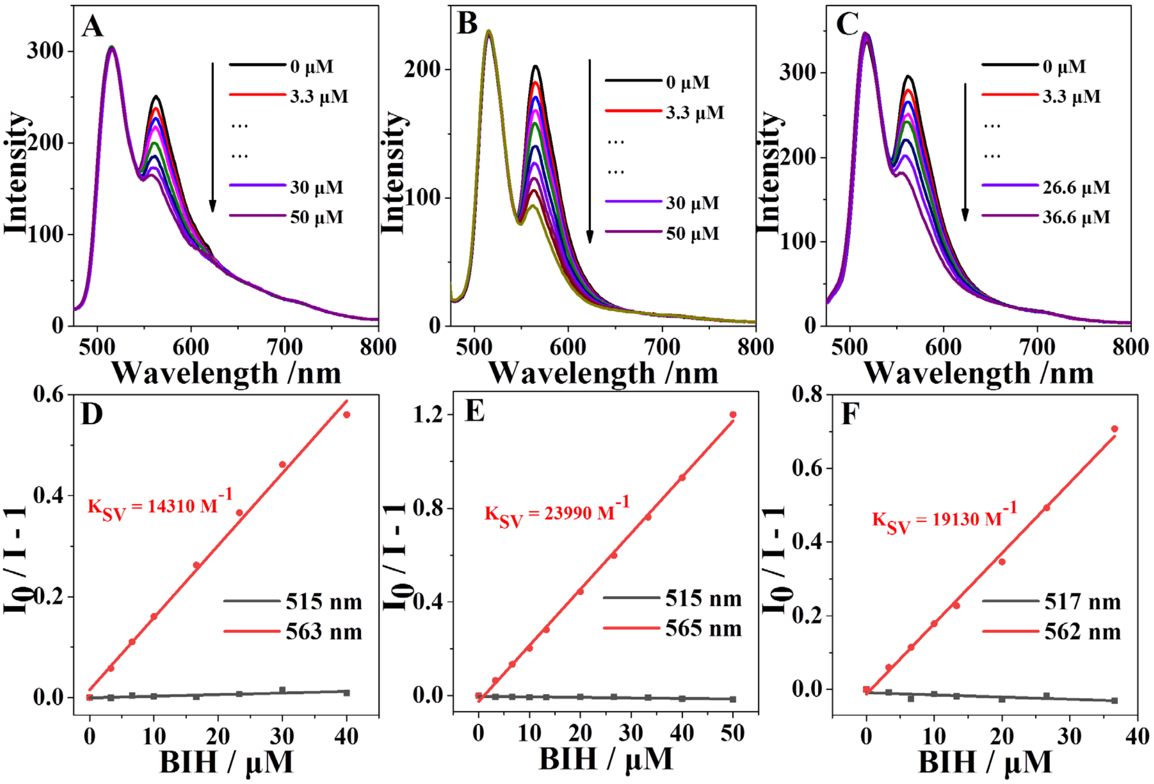

Supplementary Figure 34. FL quenching of (A) **Z-2**, (B) **Z-3**, (C) **Z-5**. with **BIH** as the quencher in acetonitrile. FL spectra of Zn PSs were performed under Ar. Stern-volmer plot of (D) **Z-2**, (E) **Z-3** and (F) **Z-5**. Condition: λ_{ex} = 455 nm, c_{ps} = 10.0 μM.

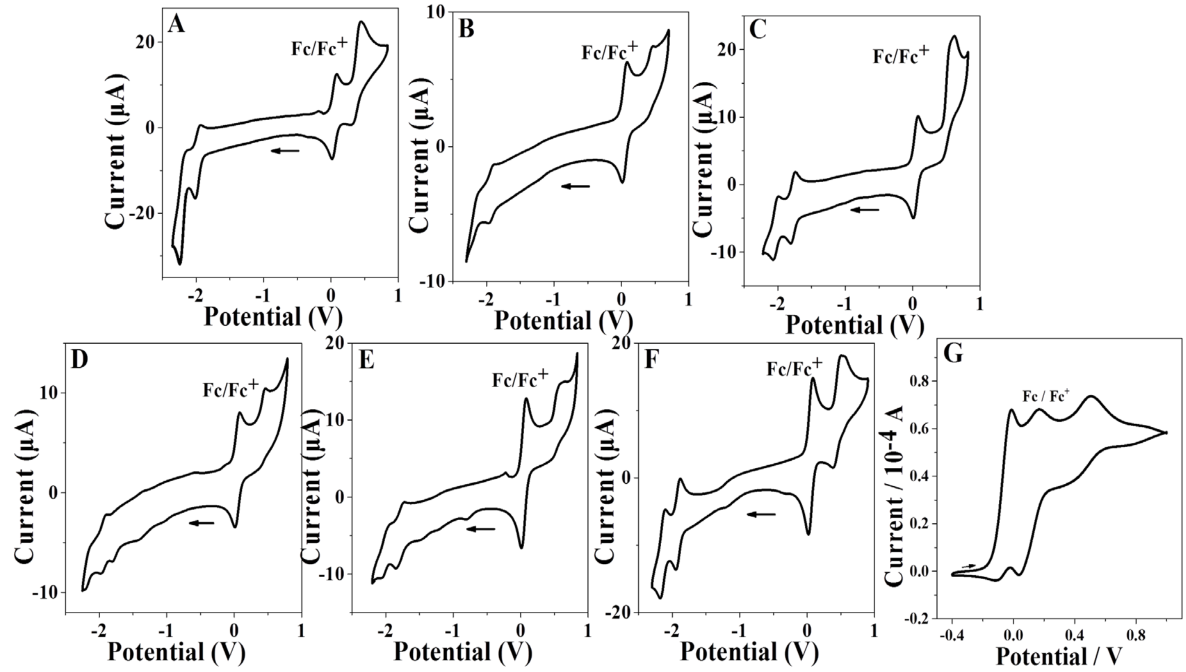

Supplementary Figure 35. CVs of (A) **Z-1**, (B) **Z-2**, (C) **Z-3**, (D) **Z-4**, (E) **Z-5**, (F) **Z-6**, (G) **BIH**. The experiments were carried out in deaerated acetonitrile solutions containing 0.5 mM photosensitizer (or BIH) with ferrocene and 0.1 M Bu_4NPF_6. Glassy carbon electrode, $Ag/AgNO_3$, and Pt silk were used as the working electrode, reference electrode, and counter electrode, respectively. Scan rate: 0.05 V/s^{-1}.

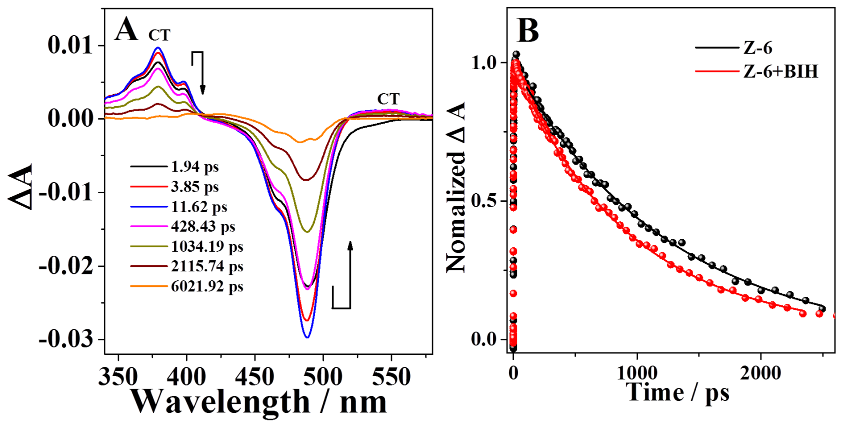

Supplementary Figure 36. Femtosecond transient absorption spectra: (A) **Z-6** and (B) Kinetic traces of **Z-6** before and after adding BIH (10 mM) followed at 379 nm. These spectra were recorded in acetonitrile upon pulsed excitation at 500 nm. c_{PS} = 10 mM.

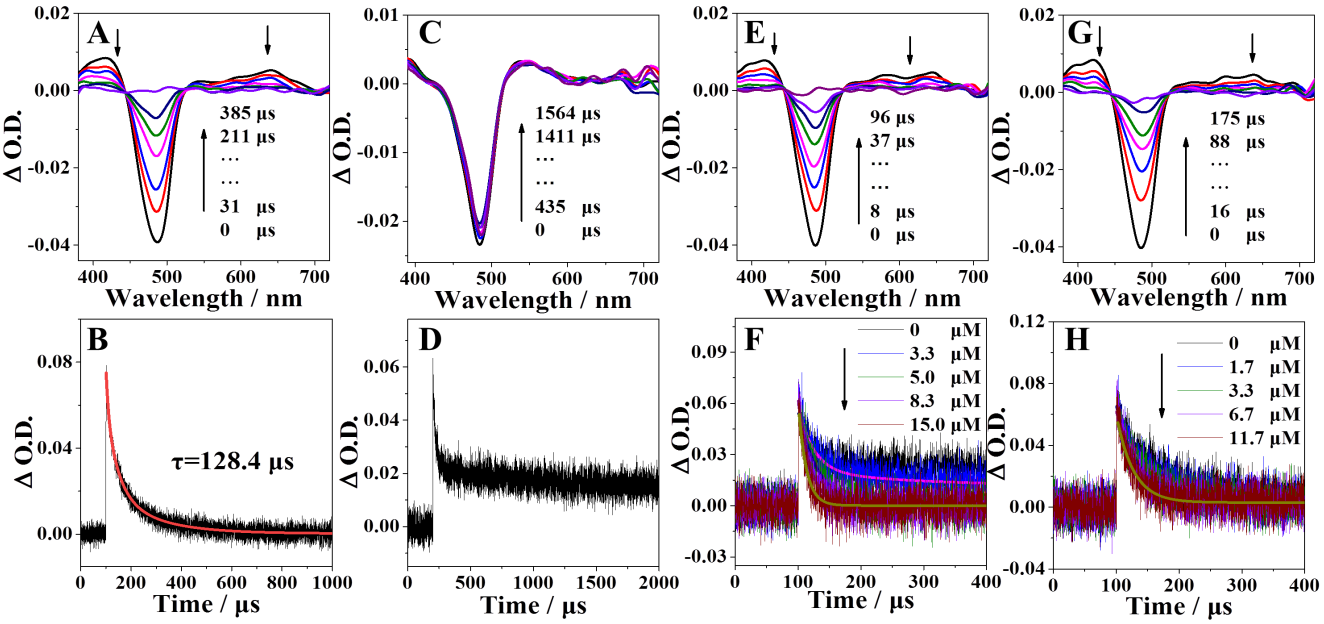

Supplementary Figure 37. Nanosecond transient absorption spectra of (A) **Z-2**, (B) the decay of **Z-2** at 490 nm, (C) **Z-2** in the presence of **BIH** (10 mM), (D) Kinetic traces of the reduced **Z-2** followed at 490 nm, (E) **Z-2** in the presence of **BIH** (10 mM) and **C-1** (15.0 μM), (*F*) Kinetic traces of **Z-2** with different concentration of **C-1** in the presence of **BIH** (10 mM) followed at 490 nm. (G) **Z-2** in the presence of **C-1** (11.7 μM), (H) Kinetic traces of **Z-2** with different concentration of **C-1** followed at 490 nm. These spectra were recorded in CH_3CN after pulsed excitation at 500 nm under Ar. c_{ps} = 10.0 μM.

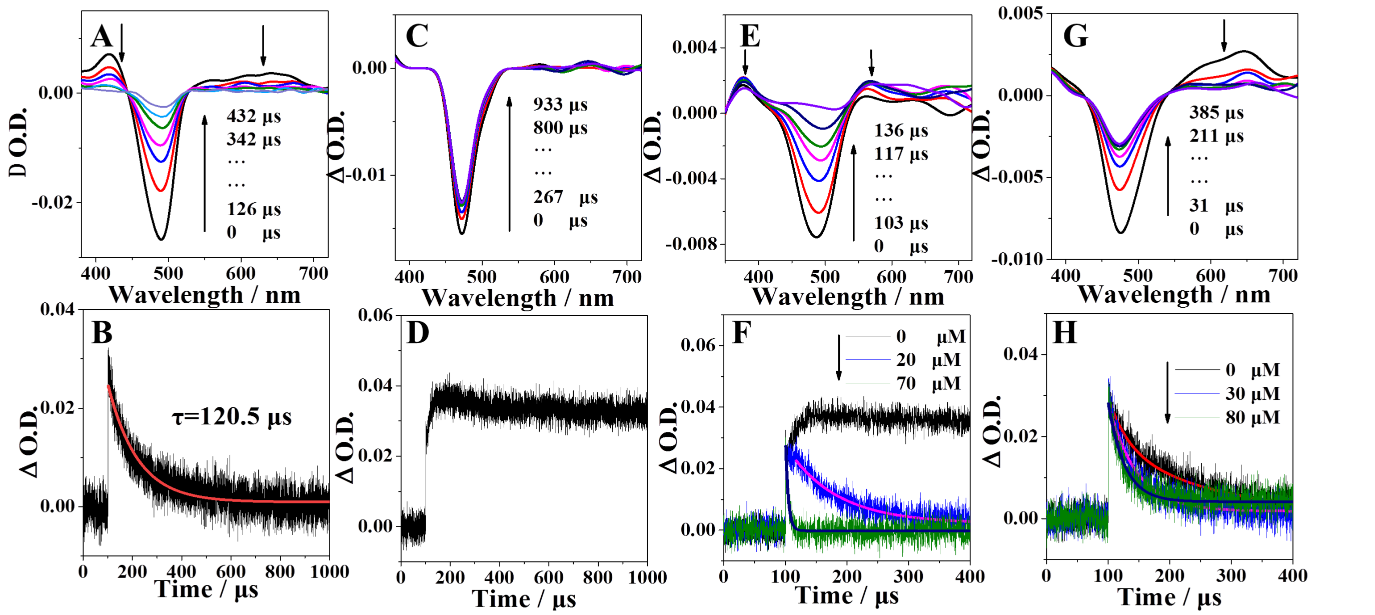

Supplementary Figure 38. Nanosecond transient absorption spectra of (A) **Z-3**, (B) the decay of **Z-3** at 490 nm, (C) **Z-3** in the presence of **BIH** (10 mM), (D) Kinetic traces of the reduced **Z-3** followed at 490 nm, (E) **Z-3** in the presence of **BIH** (10 mM) and **C-1** (70.0 µM), (F) Kinetic traces of **Z-3** with different concentration of **C-1** in the presence of **BIH** (10 mM) followed at 490 nm. (G) **Z-3** in the presence of **C-1** (80.0 µM), (H) Kinetic traces of **Z-3**with different concentration of **C-1** followed at 490 nm. These spectra were recorded in CH_3CN after pulsed excitation at 500 nm under Ar. c_{ps} = 10.0 µM.

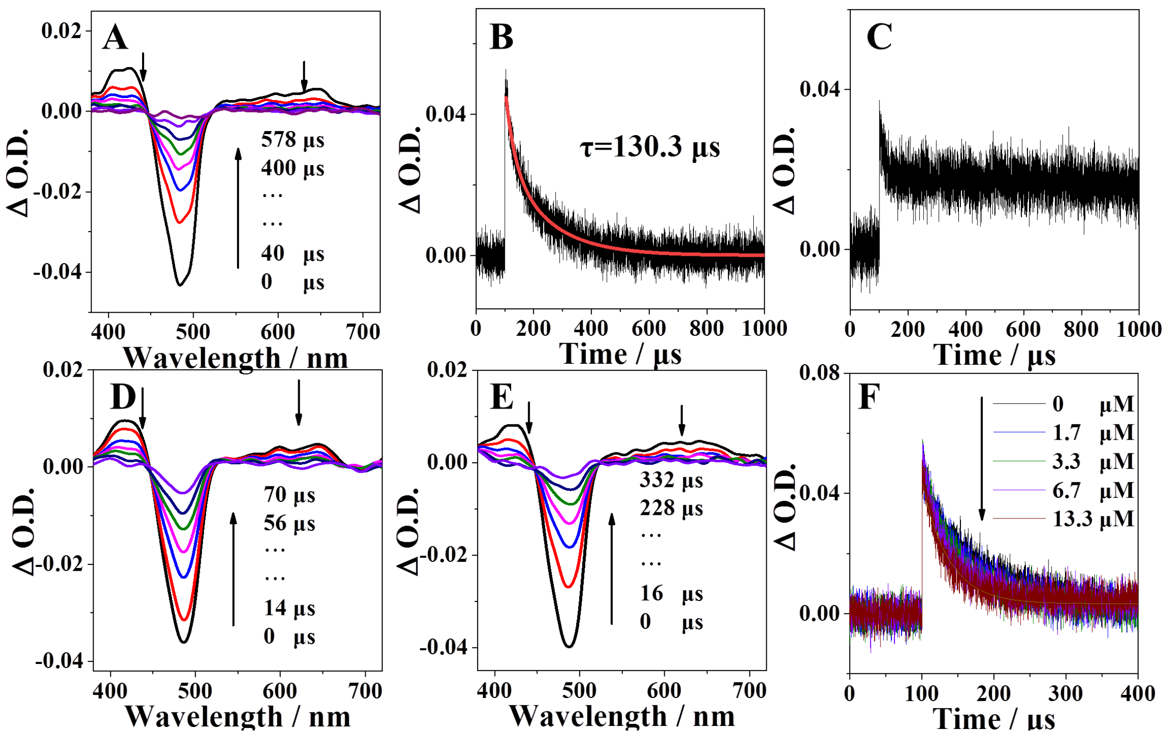

Supplementary Figure 39. Nanosecond transient absorption spectra of (A) **Z-4**, (B) the decay of **Z-4** at 490 nm, (C) Kinetic traces of the reduced **Z-4** followed at 490 nm, (D) **Z-4** in the presence of 13.3 μM of **C-1** and 10 mM **BIH**, (E) **Z-4** in the presence of 13.3 μM of **C-1**, (F) Kinetic traces of **Z-4** with different concentration of **C-1** followed at 490 nm. These spectra were recorded in CH_3CN after pulsed excitation at 500 nm under Ar. c_{ps} = 10.0 μM.

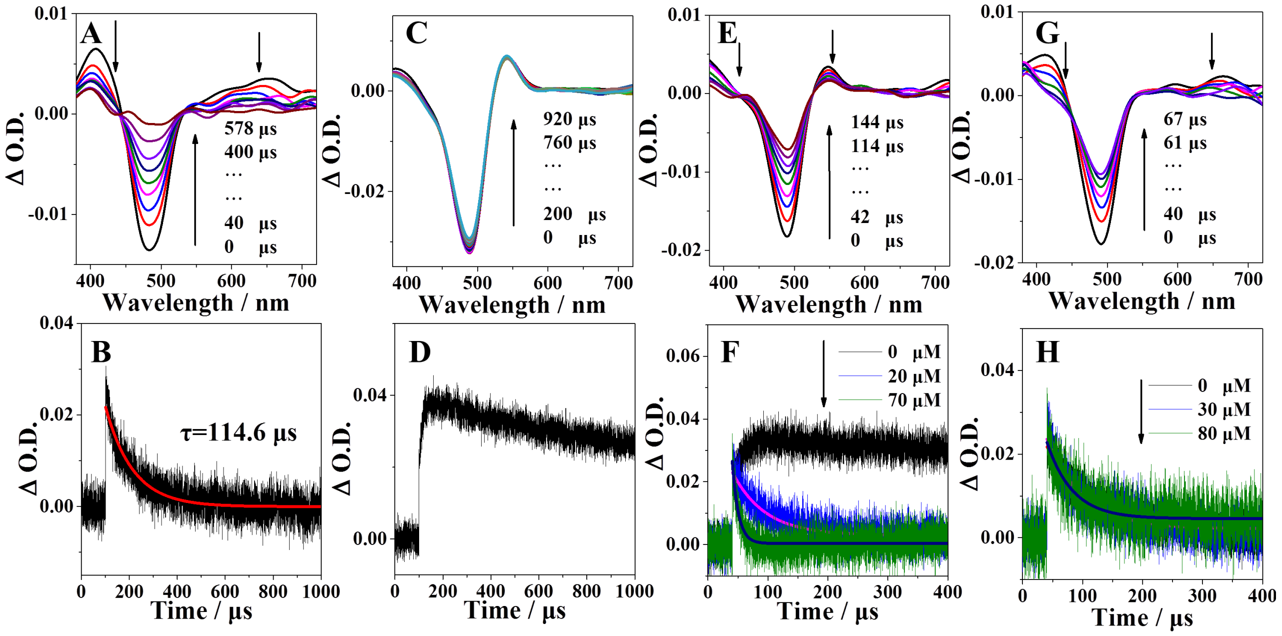

Supplementary Figure 40. Nanosecond transient absorption spectra of (A) **Z-5**, (B) the decay of **Z-5** at 490 nm, (C) **Z-5** in the presence of **BIH** (10 mM), (D) Kinetic traces of the reduced **Z-5** followed at 490 nm, (E) **Z-5** in the presence of **BIH** (10 mM) and **C-1** (70.0 μM), (F) Kinetic traces of **Z-5** with different concentration of **C-1** in the presence of **BIH** (10 mM) followed at 490 nm. (G) **Z-5** in the presence of **C-1** (80.0 μM), (H) Kinetic traces of **Z-5** with different concentration of **C-1** followed at 490 nm. These spectra were recorded in CH_3CN after pulsed excitation at 500 nm under Ar. c_{ps} = 10.0 μM.

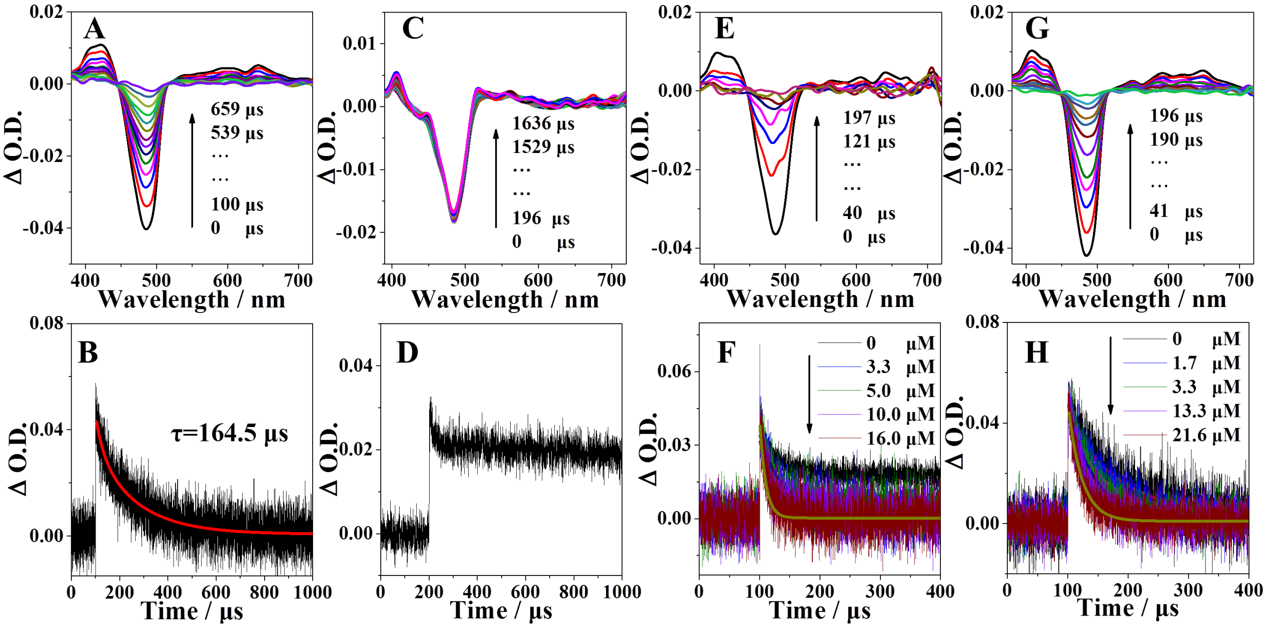

Supplementary Figure 41. Nanosecond transient absorption spectra of (A) **Z-6**, (B) the decay of **Z-6** at 490 nm, (C) **Z-6** in the presence of **BIH** (10 mM), (D) Kinetic traces of the reduced **Z-6** followed at 490 nm, (E) **Z-6** in the presence of **BIH** (10 mM) and **C-1** (16.0 μM), (F) Kinetic traces of **Z-6** with different concentration of **C-1** in the presence of **BIH** (10 mM) followed at 490 nm. (G) **Z-6** in the presence of 21.6 μM of **C-1**, (H) Kinetic traces of **Z-6** with different concentration of **C-1** followed at 490 nm. These spectra were recorded in CH_3CN after pulsed excitation at 500 nm under Ar. c_{ps} = 10.0 μM.

Supplementary Tables

Supplementary Table 1. The results of control experiments for photocatalytic CO_2 reduction towards **Z-6**.[a]

Z-6 (μM)	CO (μmol)	H_2 (μmol)	TON_{PS}	TON_{CAT}
0.5[b]	110.5	0.1	11056	553
2.5[c]	240.9	0.1	4819	1205
5.0[d]	340.8	0.2	3408	1704
20.0[e]	400.1	0.3	1000	2001

[a] Reaction conditions: **C-1** (10.0 μM), **BIH** (20.0 mM), in 20.0 mL CO_2-saturated CH_3CN / H_2O solution, Xenon lamp (＞420 nm, 80 mW•cm^{-2}), irradiation time: 8 h. [b]0.8 mM H_2O, [c]1.9 mM H_2O, [d]3.3 mM H_2O, [e]4.2 mM H_2O.

Supplementary Table 2. The results of photocatalytic CO_2 reduction.[a]

PSs	CO (μmol)	TON[b]	Φ[c]
**Z-1**	2.2	223	0.1%
**Z-2**	59.8	5981	14.1%
**Z-1**	12.7	1277	8.5%
**Z-4**	96.2	9627	21.7%
**Z-5**	11.3	1139	8.3%
**Z-6**	110.5 (51[d])	11056 (20403[d])	29.6%
**Ir-1**	32.6	3260	/
**Ru-1**	84.5	8454	/

[a]0.5 μM PSs, 10.0 μM **C-1**, 20.0 mM **BIH** and 0.8 mM H_2O in 20.0 mL CH_3CN. [b]TON values within 10 h. [c]Quantum efficiency of CO_2 reduction. Monochromator: 488 nm. c = 5.0 μM PSs, 10.0 μM **C-1**, 15.0 mM **BIH** and 3.3 mM H_2O in 5.0 mL CH_3CN. [d]0.5 μM PSs, 20.0 μM **C-1**, 40.0 mM **BIH** and 2.2 mM H_2O in 5.0 mL CH_3CN. Under CO_2 atmosphere, 300 W Xeon lamp with 420 nm filter as light source (80 mW•cm^{-2}).

Supplementary Table 3. The results of control experiments for photocatalytic CO_2 reduction.[a]

Entry	Z-6 (μM)	C-1 (μM)	BIH (mM)	CO (μmol)	H_2 (μmol)
1	0	5.0	20.0	0	0
2	5.0	0	20.0	0	0
3	5.0	5.0	0	0	0
4[b]	5.0	5.0	20.0	0	0
5[c]	5.0	5.0	20.0	0	0

[a]Reaction conditions: **Z-6** (0.5 μM), **C-1** (10.0 μM), **BIH** (20.0 mM), H_2O (0.8 mM) in 20.0 mL CO_2-saturated acetonitrile solution, Xenon lamp (> 420 nm, 80 mW•cm^{-2}), irradiation time: 8 h. [b]Without Light. [c]Ar-saturated solution.

Supplementary Table 4. Summary of photophysical data of **Z-1** – **Z-6**.

PSs	λ_{abs}[a] (nm)	ε[b] ($M^{-1}\cdot cm^{-1}$)	λ_{em}[c] (nm)	τ_F[d] (ns)	τ_T[g] (μs)	φ_F[h] (%)	φ_T[k] (%)
**Z-1**	483	126835	512	1.1[e]	5.5	<0.1[i]/0.2[j]	34
**Z-2**	488	127064	516/563	1.1[e]/1.6[f]	128.4	<0.1[i]/1.3[j]	63
**Z-3**	494	121734	515/564	1.1[e]/2.0[f]	120.5	<0.1[i]/13[j]	37
**Z-4**	488	129557	514/562	1.2[e]/2.2[f]	130.3	<0.1[i]/1.9[j]	59
**Z-5**	495	120451	518/562	1.3[e]/2.0[f]	114.6	<0.1[i]/6.9[j]	39
**Z-6**	488	127461	517	0.9[e]	164.5	<0.1[i]/2.0[j]	54

[a]10.0 μM of **Z-1** – **Z-6** in CH_3CN. [b]Molar absorption coefficient. [c]Fluorescence emission wavelength. [d]Luminescence lifetimes, λ_{ex} = 450 nm, λ_{em} = 515 nm. [e]λ_{ex} = 450 nm, λ_{em} = 515 nm. [f]λ_{ex} = 450 nm, λ_{em} = 560 nm. [g]Triplet state lifetimes, measured by transient absorption. [h]Fluorescence quantum yield. [i]In acetonitrile. [j]In toluene. [k]Triplet state quantum yield.

Supplementary Table 5. The quenching constants of PSs by BIH.

PSs	Z-2	Z-3	Z-4	Z-5
K **(M^{-1})[a]**	14310	23990	17260	19130

[a] stern-Volmer quenching constants of PSs with BIH as the quencher.

Supplementary Table 6. Redox potentials of PSs (**Z-1** – **Z-6**) and their excited state redox potentials based on triplet state are given with respect **to SCE** (Fc as internal reference, $E_{1/2}$ (Fc^+/Fc) = +0.4 V vs.SCE)

PSs	E_{ox}/V	E_{red}/V	$E_{0,0}$	*Ox_T	*Red_T
**Z-1**	0.75	-1.62	1.61	-0.86	-0.01
**Z-2**	0.77	-1.58	1.61	-0.84	0.03
**Z-3**	0.83	-1.47	1.61	-0.78	0.14
**Z-4**	0.78	-1.59	1.61	-0.83	0.02
**Z-5**	0.89	-1.45	1.61	-0.72	0.16
**Z-6**	0.81	-1.56	1.61	-0.80	0.05

*Ox_T= E_{ox}– E_{00} (where E_{ox} is the ground state oxidation potential)

*Red_T = E_{red} + E_{00} (where E_{red} is the ground state reduction potential)

The value of $E_{0,0}$ represented the optical T_1 gap.

Supplementary Table 7. Redox potentials of PSs **(Z-2 – Z-5)** and their excited state redox potentials based on charge transfer state.[a]

PSs	E_{ox}/V	E_{red}/V	E_{00}	*Ox_{CT}	*Red_{CT}
**Z-2**	0.77	-1.58	2.37	-1.60	0.79
**Z-3**	0.83	-1.47	2.36	-1.53	0.89
**Z-4**	0.78	-1.59	2.37	-1.59	0.78
**Z-5**	0.89	-1.45	2.37	-1.48	0.92

[a]Ferrocene (Fc) was used as an internal reference ($E_{1/2}$ = + 0.4 V (Fc^+/Fc) vs. SCE). A glassy carbon was used as working electrode, a platinum counter electrode and a $Ag/AgNO_3$ reference electrode.

*Ox_{CT} = E_{ox}– $E_{0,0}$ (where E_{ox} is the ground state oxidation potential)

*Red_{CT} = E_{red} + $E_{0,0}$ (where E_{red} is the ground state reduction potential)

The value of $E_{0,0}$ estimated from 5% relative intensity of CT emission.

Supplementary Table 8. Kinetic rates for intramolecular charge transfer (k_{CT}) and charge recombination (k_{rec}) of **Z-4**, **Z-4**/BIH, **Z-6** and **Z-6**/BIH in acetonitrile determined by femtosecond transient absorption measurements.

	**Z-4**	**Z-4/BIH**	**Z-6**	**Z-6/BIH**
**1/k_{CT} (ps)**	0.77	0.79	1.08	1.08
**1/k_{rec} (ns)**	1.3	0.96	1.2	0.97

References

1. Tsuchiya M, Sakamoto R and Nishihara H *et al*. Bis(dipyrrinato)zinc(II) complexes: emission in the solid state. *Inorg Chem* 2016; **54**: 5732-4.
